# Effects of *SADS‐CoV* accessory proteins NS3a, NS7a, and NS7b on viral pathogenicity: A multi‐omics investigation

**DOI:** 10.1002/imo2.70015

**Published:** 2025-04-06

**Authors:** Xiaoling Yan, Xiaoli Zhang, Ling Zhou, Xiaoya Zhao, Qianniu Li, Tian Lan, Jun Fu, Jingyun Ma

**Affiliations:** ^1^ State Key Laboratory of Swine and Poultry Breeding Industry Guangzhou China; ^2^ College of Animal Science South China Agricultural University Guangzhou China; ^3^ Shandong University–Helmholtz Institute of Biotechnology, State Key Laboratory of Microbial Technology Shandong University Qingdao China

**Keywords:** accessory protein, metabolomics, swine acute diarrhea syndrome coronavirus, transcriptomics, viral pathogenicity

## Abstract

*Swine acute diarrhea syndrome coronavirus* (*SADS‐CoV*) is an emerging, novel porcine coronavirus that causes acute diarrhea and vomiting in piglets, leading to significant neonatal morbidity and a mortality rate exceeding 90%. To date, no comprehensive studies have systematically elucidated the multi‐omics regulatory networks of the accessory proteins of *SADS‐CoV*. The role and specific functional mechanisms of the viral accessory proteins NS3a and NS7a/b in the pathogenicity of *SADS‐CoV* remain unclear. This study utilized a reverse genetics platform with *SADS‐CoV* to generate the two deletion mutant viruses *rSADS‐ΔNS3a* and *rSADS‐ΔNS7*, lacking the *NS3a* and *NS7a/b* genes, respectively. Transcriptomic and metabolomic analyses were the first‐ever performed on porcine ileal epithelial cell line IPI‐2I infected with the mutants, revealing differentially expressed genes and metabolites in comparison with the wild‐type virus. The deletion of *NS3a* and *NS7a/b* significantly impacted the expression of genes involved in amino acid metabolism (*ARG1*, *NAGS*); carbohydrate metabolism (*PCK1*); and ECM receptor interaction (*LAMB3*), with more profound effects observed with the *NS7a/b* deletion. Metabolomic analysis further confirmed that NS3a and NS7a/b primarily affect lipid, amino acid, and carbohydrate metabolism. Animal experiments in piglets demonstrated that the loss of these proteins attenuated the virulence of *SADS‐CoV*. Our findings provide insights into the pathogenic mechanisms of *SADS‐CoV* and suggest potential targets for intervention.

## Highlights

- *NS3a* and *NS7a/b* deletion attenuates *swine acute diarrhea syndrome coronavirus* (*SADS‐CoV*) virulence by disrupting host amino acid, lipid, and carbohydrate metabolism, validated through multi‐omics and piglet infection models.
- NS3a and NS7a/b modulate *IL‐11* and *LAMB3* expression, linking viral accessory proteins to inflammatory regulation and intestinal epithelial damage.
- Integrated transcriptomics‐metabolomics analysis reveals arginine biosynthesis as a key pathway influenced by NS7a/b, with α‐ketoglutarate as a central metabolic node.

## Graphical abstract

This study has elucidated the role of three *swine acute diarrhea syndrome coronavirus* (*SADS‐CoV*) accessory proteins in influencing viral pathogenicity and preliminarily explored the host molecular targets and pathways affected by the NS3a, NS7a, and NS7b proteins of *SADS‐CoV*. Our findings suggest that *SADS‐CoV* accessory proteins may modulate viral pathogenicity by affecting host metabolic pathways and immunity.

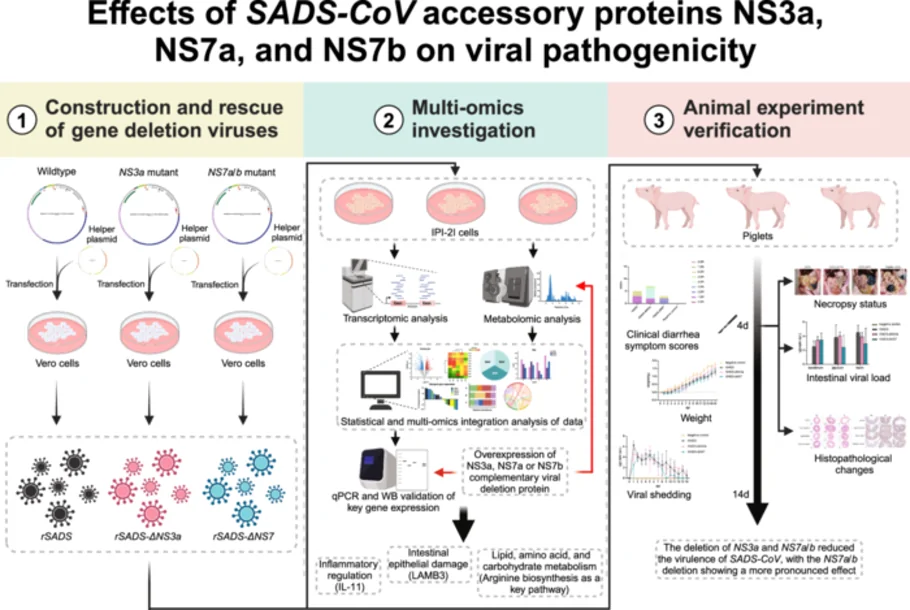

## 1 INTRODUCTION

*Swine acute diarrhea syndrome coronavirus* (*SADS‐CoV*) is a newly emerged, highly pathogenic *Alphacoronavirus* that causes acute diarrhea, vomiting, and high mortality (>90%) in piglets, with the potential for cross‐species transmission [1, 2, 3, 4, 5, 6, 7]. The viral genome encodes multiple structural proteins and accessory proteins (NS3a, NS7a/b), with the latter playing critical roles in regulating host immune responses, cell death, and viral pathogenicity [8, 9, 10]. However, significant gaps remain in understanding the functions and molecular mechanisms of *SADS‐CoV* accessory proteins, hindering the development of vaccines and optimized control strategies.

Although not essential for viral replication in vitro, coronavirus accessory proteins profoundly influence viral pathogenicity by modulating host immunity, apoptosis, and inflammatory responses [8, 9, 10]. For instance, NS3a may participate in immune evasion and cell death through ion channel activity and the NF‐κB signaling pathway [11, 12, 13, 14, 15, 16, 17, 18], while NS7a/b may antagonize interferon responses and regulate metabolic pathways [19, 20, 21]. Studies on similar proteins in other coronaviruses (e.g., *severe acute respiratory syndrome coronavirus*, *SARS‐CoV* and *porcine epidemic diarrhea virus*, *PEDV*) have shown that their deletion significantly reduces viral virulence, suggesting a central role for *SADS‐CoV* accessory proteins in pathogenic mechanisms [22, 23, 24, 25].

This study employed a reverse genetics system to construct *SADS‐CoV* accessory protein deletion mutants (*rSADS‐ΔNS3a*, *rSADS‐ΔNS7*), integrating transcriptomic and metabolomic analyses to systematically elucidate the regulatory mechanisms of NS3a and NS7a/b in viral pathogenicity. Using a cellular model of infection, we screened and validated differentially expressed genes and metabolites associated with the accessory proteins. Using an animal model of infection, we evaluated the impact of these protein gene deletions on the virulence of *SADS‐CoV*. This study aids in clarifying the virological and pathological characteristics of *SADS‐CoV*, providing crucial theoretical foundations and practical guidance for the control and prevention of, and vaccine development against, *SADS‐CoV* disease.

## 2 RESULTS

### Deletion of *SADS‐CoV NS3a* and *NS7a/b* genes does not significantly impact viral replication capacity

**FIGURE 1:**
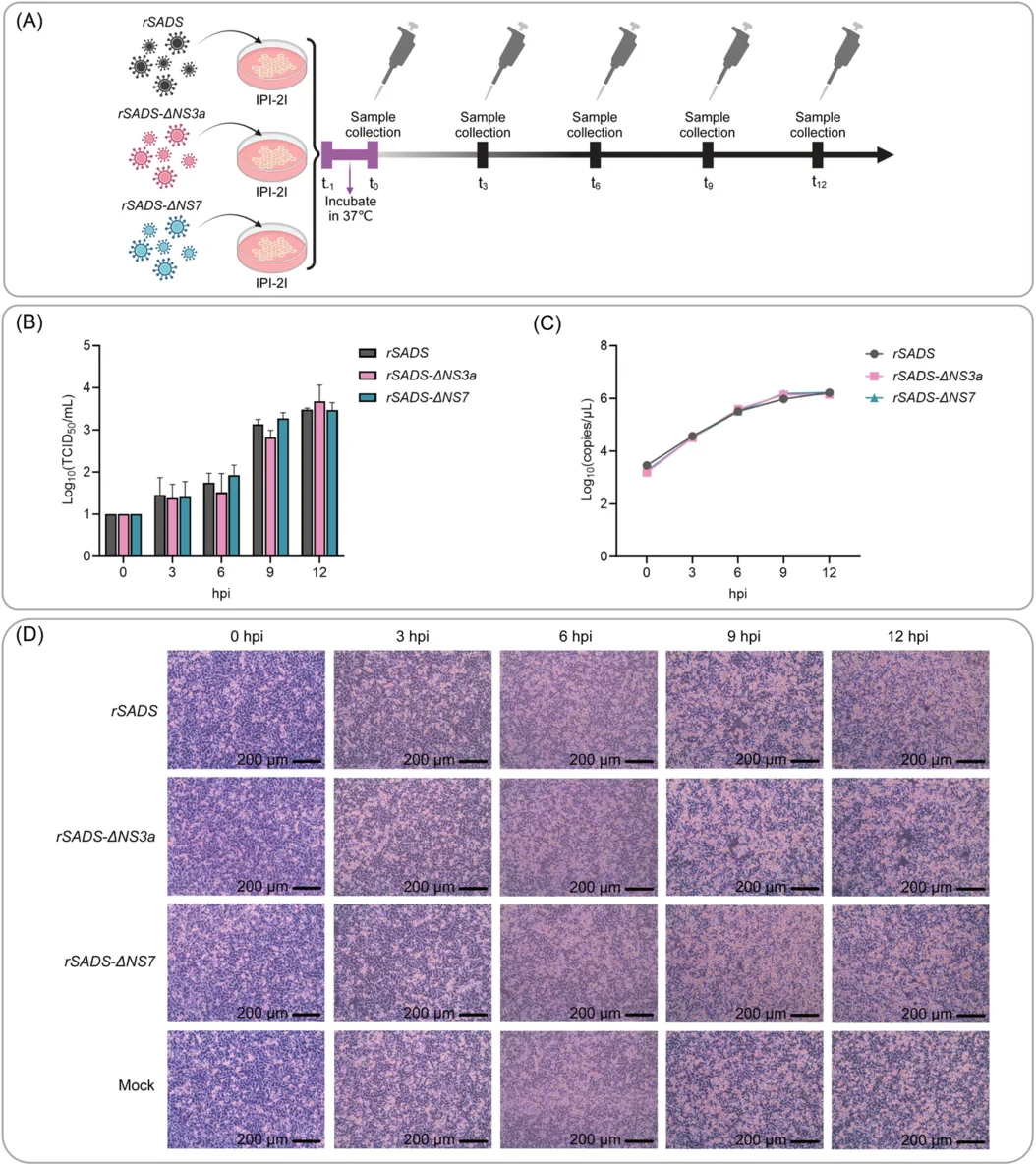
Comparison of proliferation levels of the accessory protein deletion mutant viruses and the backbone virus in IPI‐2I cells. (A) Experimental flowchart; IPI‐2I cells were infected with *rSADS‐ΔNS3a*, *rSADSΔNS7*, or *rSADS* at an 0.1 of MOI, and cell culture supernatants were collected at different time points postinfection to test virus titers (B) or to detect viral RNA copy numbers (C). (B) Virus titers were determined by infecting Vero cells in 96‐well plates. (C) Viral RNA copy numbers were determined by absolute fluorescence quantitative PCR of the viral *N* gene. Time zero indicates the first hour post infection (hpi). (D) Microscopic observation of cell lesions at different sampling time points.

The construction of the infectious clones for the *SADS‐CoV* accessory protein NS3a and NS7a/b deletion strains was completed through Red α/β recombination and Gibson assembly (Figure S1). The two recombinants were rescued by transfecting Vero cells with the final constructs (Figure S2). To determine whether the deletion of the accessory protein genes in *SADS‐CoV* affected viral replication capacity, we infected the porcine ileal epithelial cell line IPI‐2I using a multiplicity of infection (MOI) of 0.1, and supernatants were then collected at various time points to measure the viral titers of the deletion mutants and wild‐type infectious clone (Figure 1A). There were no significant differences in viral titers among the strains at any time point (Figure 1B). Additionally, viral copy numbers showed a similar trend of increasing over time by real‐time fluorescence quantitative PCR (RT‐qPCR) of the viral *N* gene, with no significant differences among the strains (Figure 1C). Under the microscope, cytopathic effects (CPE) were observed in infected cells starting at 9 h postinfection for all three viral strains. No significant differences in the extent of CPE induced by the three strains were detected (Figure 1D).

### Impact of *SADS‐CoV NS3a* and *NS7a/b* gene deletions on host metabolic and cytokine‐related pathway gene expression

**FIGURE 2:**
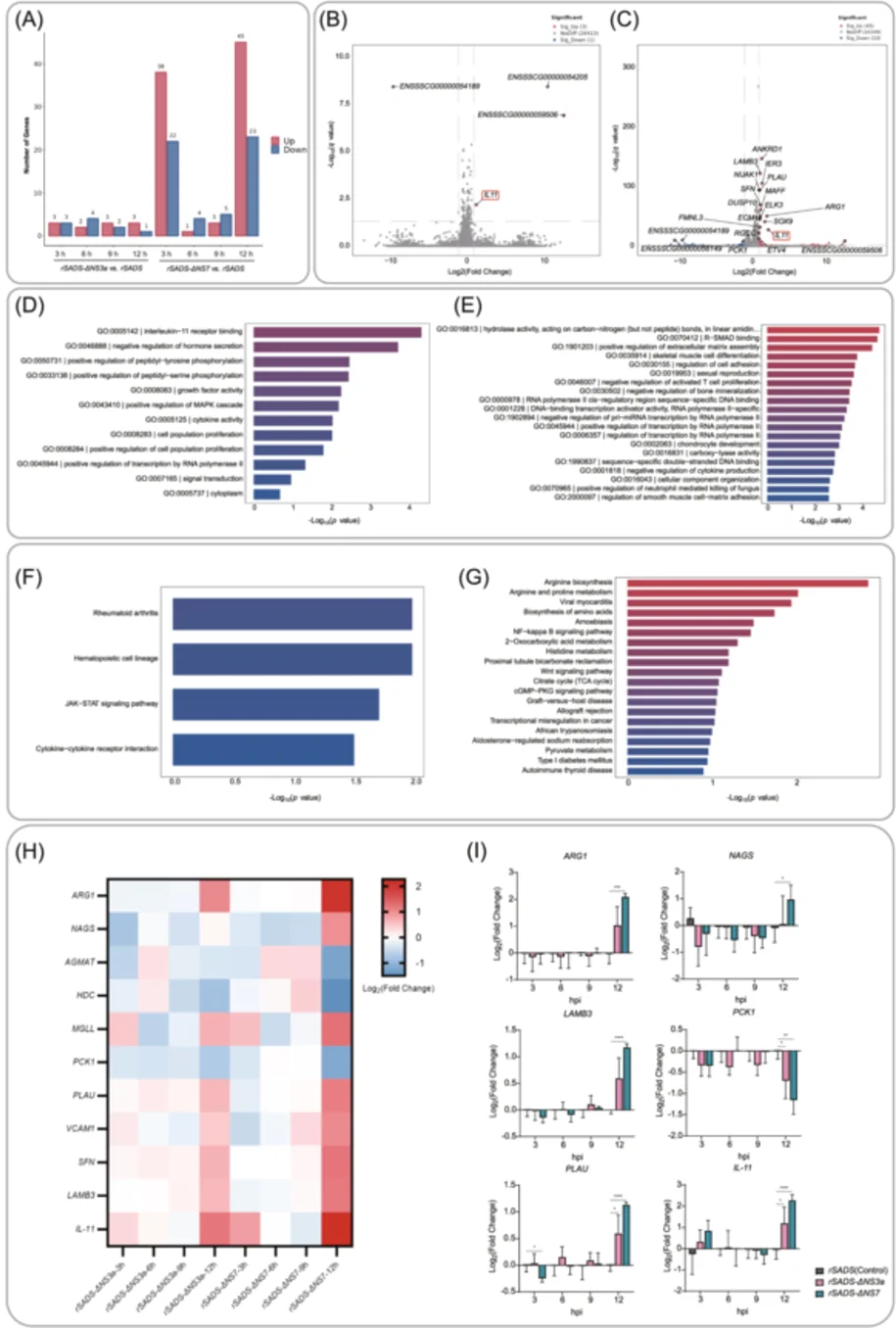
Transcriptomic analyses of IPI‐2I cells after infection with the accessory protein deletion mutant viruses and the backbone virus. (A) Number of DEGs in each comparison group. Red indicates upregulation; blue indicates downregulation. Selection criteria are *q* value < 0.05 and |log_2_(fold change)| > 1. Volcano plots of DEGs in the (B) *rSADS‐ΔNS3a* infection group compared to the *rSADS* infection group and (C) *rSADS‐ΔNS7* infection group compared to the *rSADS* infection group at 12 h postinfection. Significantly DEGs are color‐coded: red represents upregulated DEGs, blue indicates downregulated DEGs, while gray dots correspond to nonsignificant genes. GO enrichment analysis for the (D) *rSADS‐ΔNS3a*‐12h versus *rSADS*‐12h comparison group and (E) *rSADS‐ΔNS7*‐12h versus *rSADS*‐12h comparison group. The 20 most significantly enriched GO terms (ranked by ascending *p* values) were visualized in a horizontal bar plot, with GO terms labeled on the *y*‐axis and the −Log_10_(*p*‐value) of enrichment significance on the *x*‐axis. Smaller *p* values (larger −Log_10_(*p*‐value) values) reflect greater enrichment significance. KEGG enrichment analysis for the (F) *rSADS‐ΔNS3a*‐12h versus *rSADS*‐12h comparison group and (G) *rSADS‐ΔNS7*‐12h versus *rSADS*‐12h comparison group. The 20 most significantly enriched KEGG pathways (ranked by *p*‐value) were visualized in a horizontal bar plot, with pathway labels on the *y*‐axis and −Log_10_(*p*‐value) (representing enrichment significance) on the *x*‐axis, where lower *p* values result in larger *x*‐axis values denoting stronger statistical enrichment. (H) Heatmap of relative expression levels of major DEGs. Red indicates upregulation; blue indicates downregulation. (I) Bar graph of relative expression levels of the DEGs *ARG1*, *NAGS*, *PCK1*, *LAMB3*, *PLAU*, and *IL‐11*. hpi is the abbreviation for hours post infection. *p < 0.05; **p < 0.01; ***p < 0.001; ****p < 0.0001.

To investigate the impact of the deletion of the *SADS‐CoV* accessory protein genes on various biological processes and gene expression in host cells, and to identify potential pathways and molecular targets affected by these proteins, we utilized the recombinant viruses *rSADS*, *rSADS‐ΔNS3a*, and *rSADS‐ΔNS7* to infect IPI‐2I cells at an MOI of 0.1, followed by transcriptome sequencing. The results showed that the *rSADS‐ΔNS3a* infection group, compared to the *rSADS* group, had 6, 6, 5, and 4 differentially expressed genes (DEGs) at 3, 6, 9, and 12 h, respectively (Figure 2A); after filtering out data with FPKM (fragments per kilobase of exon model per million mapped fragments) of less than 1 and unannotated data, only one DEG remained, that is, *interleukin‐11* (*IL‐11*), which showed increased expression at 12 h (Figure 2B). In the *rSADS‐ΔNS7* infection group, compared to the *rSADS* group, 60, 5, 8, and 68 DEGs were identified at 3, 6, 9, and 12 h, respectively (Figure 2A), with 20 DEGs remaining after similar filtering, mostly showing increased expression, including *IL‐11* (Figure 2C). Therefore, *IL‐11* was a common DEG in both the *rSADS‐ΔNS3a* and *rSADS‐ΔNS7* infection groups compared to the control, and this gene was upregulated in both cases.

In the *rSADS‐ΔNS3a*‐12h versus *rSADS*‐12h comparison group, the most significantly enriched gene ontology (GO) term was IL‐11 receptor binding molecular function, followed by negative regulation of hormone secretion biological process (Figure 2D). The *rSADS‐ΔNS7*‐12h versus *rSADS*‐12h comparison group showed significant enrichment for hydrolase activity acting on carbon‐nitrogen (but not peptide) bonds molecular function, followed by R‐SMAD binding molecular function and positive regulation of extracellular matrix (ECM) assembly biological process (Figure 2E). Four pathways were enriched in the *rSADS‐ΔNS3a*‐12h versus *rSADS*‐12h KEGG analysis, including rheumatoid arthritis, hematopoietic cell lineage, JAK‐STAT signaling pathway, and cytokine‐cytokine receptor interaction, with rheumatoid arthritis and hematopoietic cell lineage being the most significantly enriched, although the JAK‐STAT signaling pathway and cytokine‐cytokine receptor interaction were more relevant to our research focus (Figure 2F). In the comparison group *rSADS‐ΔNS7*‐12h versus *rSADS*‐12h, the most significant KEGG enrichment was observed in arginine biosynthesis, followed by arginine and proline metabolism, viral myocarditis, biosynthesis of amino acids, amoebiasis, NF‐κB signaling pathway, and 2‐oxocarboxylic acid metabolism. Among the top 20 KEGG entries with the smallest *p* values, 7 were related to metabolic pathways. Therefore, the subsequent analysis focused on metabolism‐related molecular pathways (Figure 2G).

To visually compare the differential expression of genes between comparison groups, we selected representative DEGs. The metabolism‐related genes *arginase 1 (ARG1)*, *N‐acetylglutamate synthetases (NAGS)*, and *monoacylglycerol lipase (MGLL)* showed upregulated expression at 12 h postinfection in both mutants (Figure 2H), whereas the *agmatine ureohydrolase (AGMAT)*, *histidine decarboxylase (HDC)*, and *phosphoenolpyruvate carboxykinase 1* (*PCK1*) genes showed downregulated expression. Among the encoded proteins, PCK1 is the rate‐limiting enzyme in the regulation of gluconeogenesis and is involved in the maintenance of blood glucose levels and a wide range of metabolic and biological processes, including glucose metabolism, lipid metabolism, aging, diabetes, and tumor cell proliferation and apoptosis. Genes related to the NF‐κB signaling pathway, *plasminogen activator, urokinase (PLAU)*, *vascular cell adhesion protein 1 (VCAM1)*, and the p53 signaling pathway‐related ‌protein *stratifin (SFN)*, as well as the ECM receptor interaction‐related gene *laminin beta 3* (*LAMB3*), and the cytokine gene *IL‐11* were all upregulated. The trends in gene expression increases and decreases were consistent across the comparison groups *rSADS‐ΔNS3a*‐12h versus *rSADS*‐12h and *rSADS‐ΔNS7*‐12h versus *rSADS*‐12h (Figure 2H). Bar charts of the expression levels of the DEGs *ARG1*, *NAGS*, *PCK1*, *LAMB3*, *PLAU*, and *IL‐11* at different time points were constructed and further demonstrated that the *rSADS‐ΔNS3a*‐12h versus *rSADS*‐12h and *rSADS‐ΔNS7*‐12h versus *rSADS*‐12h comparison groups showed differences in the expression of these genes at 12 h postinfection, with differences in the *rSADS‐ΔNS7*‐12h versus *rSADS*‐12h group being more pronounced, although the trends in expression increases and decreases were consistent across both comparison groups (Figure 2I).

**FIGURE 3:**
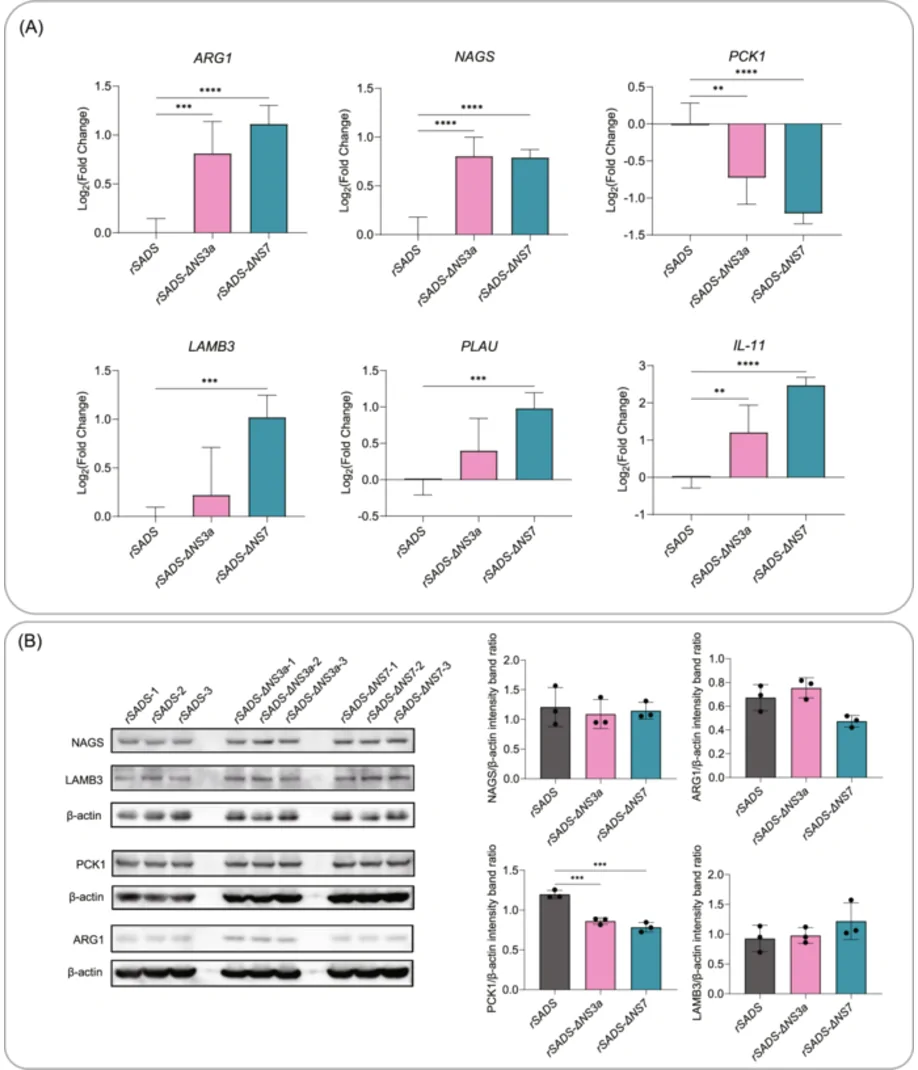
Confirmation of mRNA and protein expression levels of DEGs in IPI‐2I cells after infection. (A) RT‐qPCR confirmation of mRNA levels of the DEGs *ARG1*, *NAGS*, *PCK1*, *LAMB3*, *PLAU*, and *IL‐11*. Gene expression levels were calculated using the 2^−ΔΔCt^ method, *rSADS* group was used as a control group, and *β‐actin* as an endogenous reference. (B) Western blot confirmation of protein expression levels of the DEGs ARG1, NAGS, PCK1, and LAMB3. All experiments were repeated at least three times.

To validate the results obtained from transcriptomic sequencing, we employed RT‐qPCR and western blot analysis to measure the mRNA and protein expression levels of the selected genes. The RT‐qPCR results (Figure 3A) were consistent with the transcriptomic sequencing findings, with significant differences in the expression levels of *ARG1*, *NAGS*, *PCK1*, *LAMB3*, *PLAU*, and *IL‐11* across both comparison groups at 12 h postinfection, with more pronounced differences in the *rSADS‐ΔNS7*‐12h versus *rSADS*‐12h comparison group, although with consistent trends in expression across both comparison groups.

Analysis of the protein expression levels of ARG1, NAGS, PCK1, and LAMB3 by western blot and densitometry revealed significant differences in the expression of the PCK1 protein among the comparison groups, with the protein expression trends corresponding directly with the mRNA expression trends (Figure 3B).

Overall, our results indicate that the absence of the *SADS‐CoV* accessory protein NS3a significantly affected the transcription levels of the metabolism‐related genes *ARG1*, *NAGS*, and *PCK1*, and the cytokine gene *IL‐11*, with significant differences also observed in the protein expression level of PCK1. Similarly, the absence of the accessory proteins NS7a/b led to significant changes in the transcription levels of *ARG1*, *NAGS*, and *PCK1*, the NF‐κB signaling pathway‐related gene *PLAU*, the ECM receptor interaction‐related gene *LAMB3*, and *IL‐11*, as well as significant changes in the protein expression level of PCK1.

### Impact of *SADS‐CoV* accessory protein gene deletion on host fatty acid, amino acid, and carbohydrate metabolism

**FIGURE 4:**
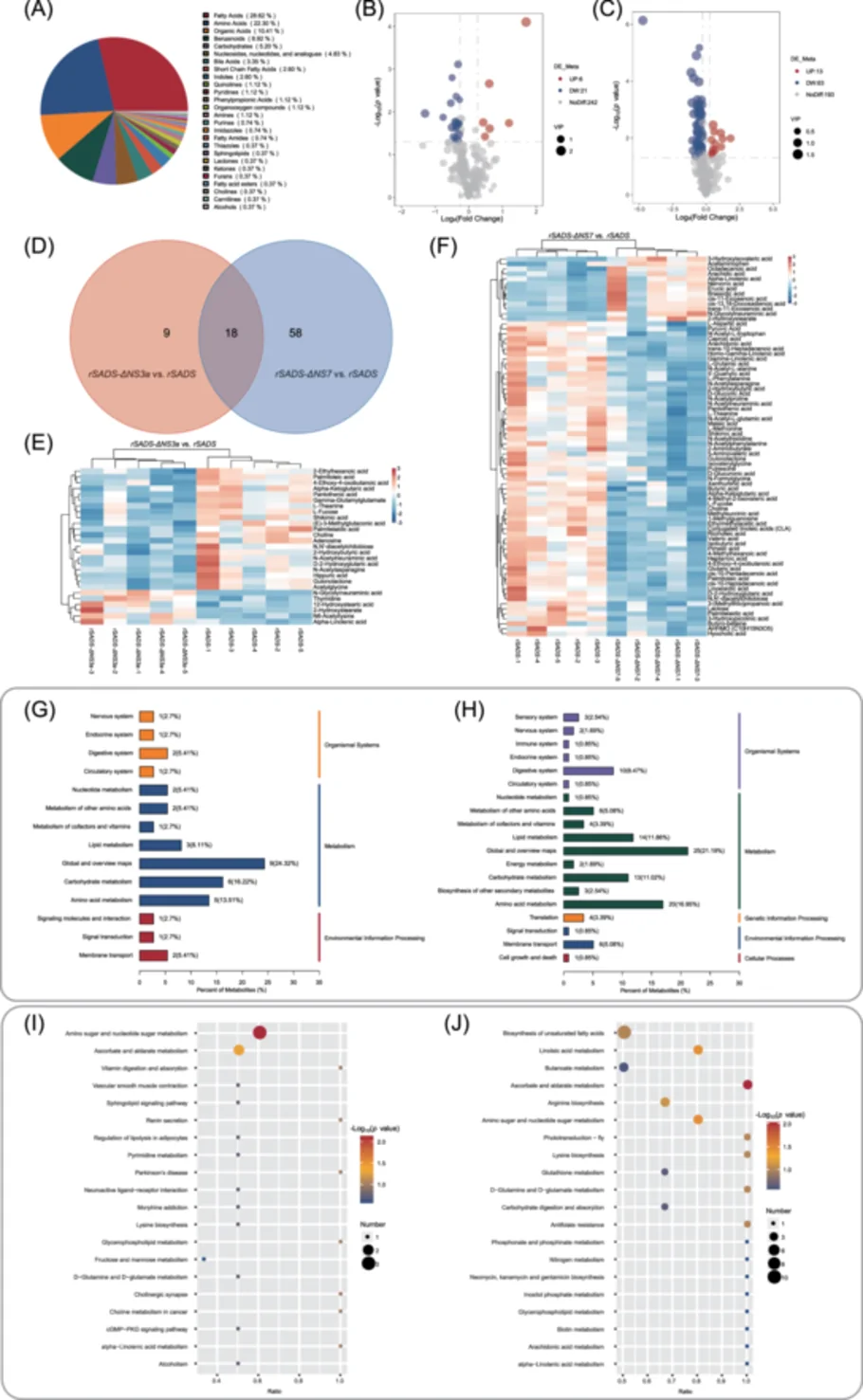
Metabolomic analyses of IPI‐2I cells after infection with the accessory protein deletion mutant viruses and the backbone virus. (A) Chemical classification statistics of identified metabolites. Volcano plots of differentially expressed metabolites in the (B) *rSADS‐ΔNS3a* infection group compared to the *rSADS* infection group and (C) *rSADS‐ΔNS7* infection group compared to the *rSADS* infection group at 12 h postinfection. Red indicates upregulation; blue indicates downregulation. Selection criteria were VIP > 1.0, FC > 1.2 or FC < 0.833, and *p*‐value < 0.05. (D) Venn diagram analysis of differentially expressed metabolites between comparison group *rSADS‐ΔNS3a* versus *rSADS* and *rSADS‐ΔNS7* versus *rSADS*. Clustering analysis of differentially expressed metabolites for the comparison group (E) *rSADS‐ΔNS3a* versus *rSADS* and (F) *rSADS‐ΔNS7* versus *rSADS*. Vertical clustering represents samples (numbered 1 to 5), horizontal clustering represents metabolites, and shorter branches indicate higher similarity. AHHMO (C10H15N3O5) is a custom abbreviation for 4‐amino‐1‐[4‐hydroxy‐5‐(hydroxymethyl)‐3‐methoxy‐2‐oxolanyl]‐2‐pyrimidinone. (G, H) KEGG classification chart for the comparison group (G) *rSADS‐ΔNS3a* versus *rSADS* and (H) *rSADS‐ΔNS7* versus *rSADS*. The horizontal axes represent the percentage of metabolites annotated to a specific KEGG pathway out of all annotated metabolites. The vertical axes show the primary classification of the KEGG pathway on the right and the secondary classification on the left. (I, J) KEGG enrichment bubble diagrams for the comparison group (I) *rSADS‐ΔNS3a* VS *rSADS* and (J) *rSADS‐ΔNS7* VS *rSADS*. The horizontal axes represent *x*/*y* (the number of differential metabolites in the corresponding metabolic pathway/the total number of identified metabolites in that pathway). A larger value indicates a higher degree of enrichment of differential metabolites in that pathway. The color of the points represents the *p*‐value from the hypergeometric test, with smaller values indicating greater reliability and statistical significance. The size of the points represents the number of differential metabolites in the corresponding pathway, with larger points indicating more differential metabolites within that pathway.

Based on the KEGG enrichment analysis of the gene expression profiles, we conducted a targeted metabolomics of biomarkers associated with the relevant metabolic pathways to verify the effects of the *SADS‐CoV* accessory protein gene deletion on host metabolic processes and metabolite expression, and to further elucidate the functional mechanisms of these proteins, metabolomic analysis was employed using infected IPI‐2I cells. The results identified 269 types of metabolites across all three test groups (two mutants and control virus infection), with fatty acids, amino acids, and organic acids constituting, respectively, 28.62%, 22.30%, and 10.41% of the total (Figure 4A). Using thresholds of variable importance in the projection (VIP) > 1.0, fold change (FC) > 1.2 or FC < 0.833, and *p* value < 0.05, the *rSADS‐ΔNS3a* infection group had 27 differential metabolites when compared to the *rSADS* group, with 6 significantly upregulated and 21 significantly downregulated (Figure 4B); the *rSADS‐ΔNS7* infection group exhibited 76 differential metabolites, with 13 significantly upregulated and 63 significantly downregulated (Figure 4C). Thus, the deletion of the *NS7a/b* genes appears to have had a greater impact on metabolic processes than did the deletion of *NS3a*.

A Venn diagram was used to display the shared and unique differential metabolites among the groups, and the results indicated that the *rSADS‐ΔNS3a* versus *rSADS* comparison group and the *rSADS‐ΔNS7* versus *rSADS* comparison group shared 18 differential metabolites, accounting for 67% of the total differential metabolites in the *rSADS‐ΔNS3a* group and 24% in the *rSADS‐ΔNS7* group. Therefore, the metabolic impact of the *NS3a* deletion largely aligned with that of *NS7a/b* deletion, but the latter affected a broader range of metabolic processes (Figure 4D).

Cluster analysis was performed to evaluate the metabolic patterns under different experimental conditions. Metabolites with similar metabolic patterns might share similar functions or participate in the same metabolic process or cellular pathway, and by clustering metabolites with similar or close metabolic patterns, the potential functions of some metabolites can be inferred. Hierarchical cluster analysis of the differential metabolites from both groups allowed the detection of differences in metabolic expression patterns between and within groups. The results, as shown in Figure 4E,F, demonstrate distinct clustering and good reproducibility within groups. In the *rSADS‐ΔNS3a* versus *rSADS* comparison, 27 differential metabolites were identified, including 8 fatty acids, 5 amino acids, and 4 carbohydrates, whereas 76 differential metabolites were identified in the *rSADS‐ΔNS7* versus *rSADS* comparison, including 32 fatty acids, 16 amino acids, and 7 carbohydrates. Therefore, the accessory proteins NS3a and NS7a/b primarily affect the metabolism of fatty acids, amino acids, and carbohydrates, with the deletions of *NS7a/b* having a greater impact on these metabolic processes.

KEGG pathway analysis results showed that the *rSADS‐ΔNS3a* versus *rSADS* comparison group was mainly enriched in pathways related to metabolism, with a specific focus on pathways included in the category of global and overview maps, carbohydrate metabolism, and amino acid metabolism (Figure 4G). Among them, the pathway with the most enriched differential metabolites was amino sugar and nucleotide sugar metabolism (Figure 4I). The *rSADS‐ΔNS7* versus *rSADS* comparison group was also primarily enriched in metabolic pathways, particularly in global and overview maps, amino acid metabolism, lipid metabolism, and carbohydrate metabolism (Figure 4H), with the biosynthesis of unsaturated fatty acids as the pathway with the most enriched differential metabolites (Figure 4J). These results further confirm that accessory proteins NS3a and NS7a/b significantly influence lipid, amino acid, and carbohydrate metabolic processes.

Overall, the deletion of the genes encoding the *SADS‐CoV* accessory proteins NS3a and NS7a/b significantly impacted fatty acid metabolism, amino acid metabolism, and carbohydrate metabolism, with the effects of the *NS7a/b* deletion being more pronounced on these metabolic processes.

### Multi‐omics integration analysis

**FIGURE 5:**
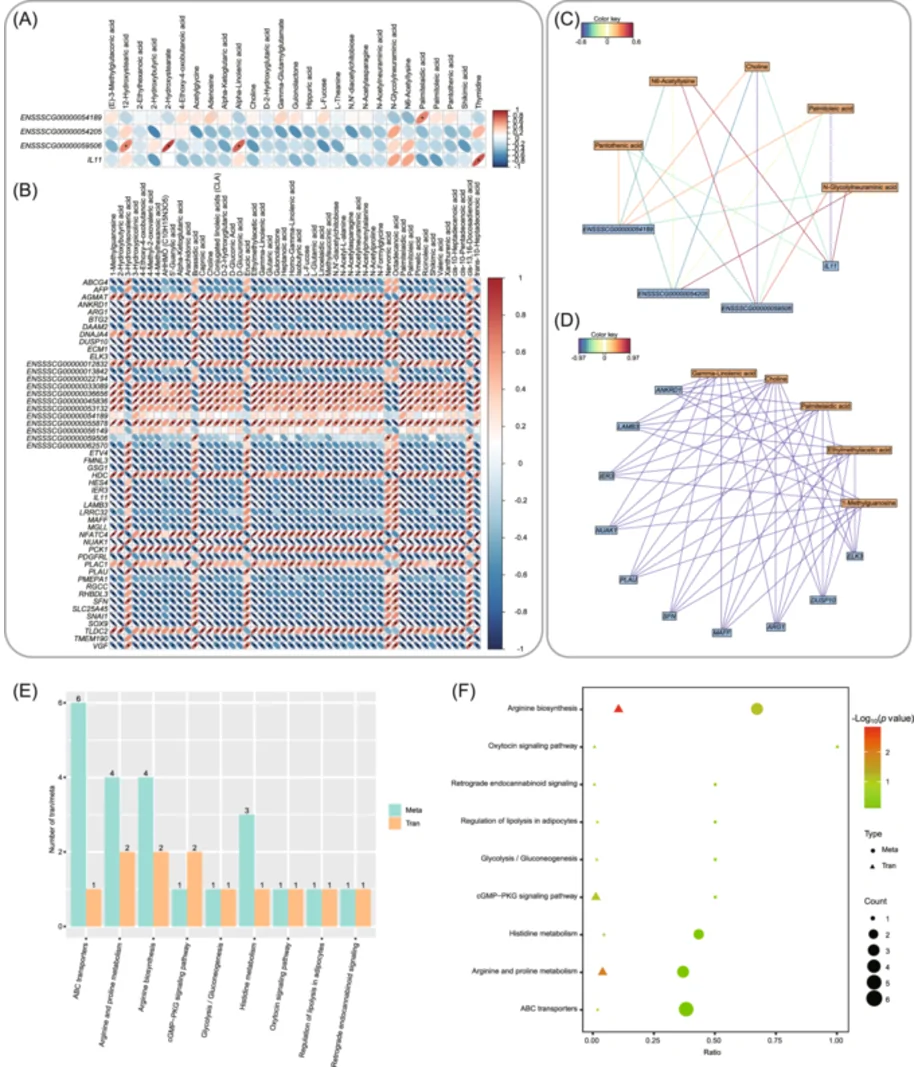
Multi‐omics integration analysis. (A, B) Heatmaps of the correlation analysis between differential metabolites and differential gene expression in the comparison group (A) *rSADS‐ΔNS3a* versus *rSADS* and (B) *rSADS‐ΔNS7* versus *rSADS*. The vertical axes represent differential metabolites, the horizontal axes represent DEGs blue indicates a negative correlation, and red indicates a positive correlation. AHHMO (C10H15N3O5) is a custom abbreviation for 4‐amino‐1‐[4‐hydroxy‐5‐(hydroxymethyl)‐3‐methoxy‐2‐oxolanyl]‐2‐pyrimidinone. Network diagram of the correlation between differential metabolites and differential gene expression in the comparison group (C) *rSADS‐ΔNS3a* versus *rSADS* and (D) *rSADS‐ΔNS7* versus *rSADS*. Squares represent metabolites (orange squares) and genes (blue squares). The colors of the lines indicate the correlation, with red for positive correlation, blue for negative correlation, and a deeper color indicating higher correlation coefficient. (E) KEGG pathway distribution statistics of DEGs and differential small molecule metabolites in the comparison group *rSADS‐ΔNS7* versus *rSADS*. (F) KEGG enrichment bubble chart of DEGs and differential metabolites in the comparison group *rSADS‐ΔNS7* versus *rSADS*. The horizontal axis shows the ratio of the number of differential metabolites or DEGs enriched in the pathway to the number of annotated metabolites or genes in the pathway (ratio); vertical axis shows the KEGG pathways co‐enriched in the metabolomics‐transcriptomics analysis; “Count” indicates the number of enriched metabolites or genes in the pathway; and color represents the *p*‐value, with the brighter the color, the smaller the *p*‐value and the more significant the pathway enrichment.

A correlation analysis was conducted between the genes that were significantly differentially expressed in the transcriptomic analysis and the significantly differentially expressed metabolites obtained from the metabolomic analysis at 12 h postinfection, using the Pearson correlation coefficient to measure the degree of association between the differential genes and differential metabolites. A correlation coefficient less than 0 indicates a negative correlation, while a coefficient greater than 0 indicates a positive correlation. The results showed a significant positive correlation between *IL‐11* and the metabolite thymidine in the comparison group *rSADS‐ΔNS3a* versus *rSADS* (Figure 5A). In the comparison group *rSADS‐ΔNS7* versus *rSADS*, most DEGs had significant correlations with most differential metabolites. Specifically, *ARG1*, *IL‐11*, *LAMB3*, and *PLAU* were significantly negatively correlated with most differential metabolites, whereas *PCK1*, *HDC*, and *NFATC4* were significantly positively correlated with most differential metabolites (Figure 5B). Network graphs were used to visually represent the relationships between metabolites and genes, and the top 10 differential genes and the top 5 differential metabolites were selected for plotting, with metabolites marked in orange and genes in blue, and correlations between them indicated by straight lines (Figure 5C,D). The results showed that, in the comparison group *rSADS‐ΔNS3a* versus *rSADS*, *IL‐11* was positively correlated with N‐glycolylneuraminic acid and N6‐acetyllysine, and negatively correlated with pantothenic acid, choline, and palmitoleic acid (Figure 5C). In the comparison group *rSADS‐ΔNS7* versus *rSADS*, the DEGs were negatively correlated with the differential metabolites (Figure 5D).

All identified differential genes and metabolites were mapped to the KEGG pathway database to obtain their common pathway information and to identify the main biochemical and signal transduction pathways that involved both differential metabolites and genes. The results showed no common enriched pathways for the differential genes and metabolites in the comparison group *rSADS‐ΔNS3a* versus *rSADS*. However, in the comparison group *rSADS‐ΔNS7* versus *rSADS*, DEGs and differential metabolites were primarily enriched in pathways such as ABC transporters, arginine and proline metabolism, and arginine biosynthesis (Figure 5E). Among these pathways, the most significantly enriched pathway was arginine biosynthesis, which includes the genes *ARG1* and *NAGS*, as well as the metabolites l‐aspartic acid, N‐acetyl‐l‐glutamic acid, alpha‐ketoglutaric acid, and l‐glutamic acid (Figure 5F). These commonly enriched pathways were also mapped using iPath (interactive Pathways Explorer, https://pathways.embl.de), revealing that the differential genes and metabolites were jointly enriched in processes such as amino acid metabolism, carbohydrate metabolism, metabolism of other secondary metabolites, and metabolism of other amino acids (Figure S3).

### Overexpression of missing proteins compensates for differences in gene and metabolite expression levels

To test whether overexpressing the missing viral accessory proteins could eliminate the differences in mRNA transcription and protein expression observed between the deletion mutant viruses (*rSADS‐ΔNS3a* and *rSADS‐ΔNS7*) and the parental virus *rSADS*, we constructed overexpression plasmids for *NS3a*, *NS7a*, and *NS7b*. The expression levels of the Flag‐tagged proteins at the transcriptional and protein levels were verified using RT‐qPCR, western blot, and immunofluorescence assay (IFA), with the results showing that all three proteins were expressed at both the mRNA and protein levels (Figure S4A–C).

**FIGURE 6:**
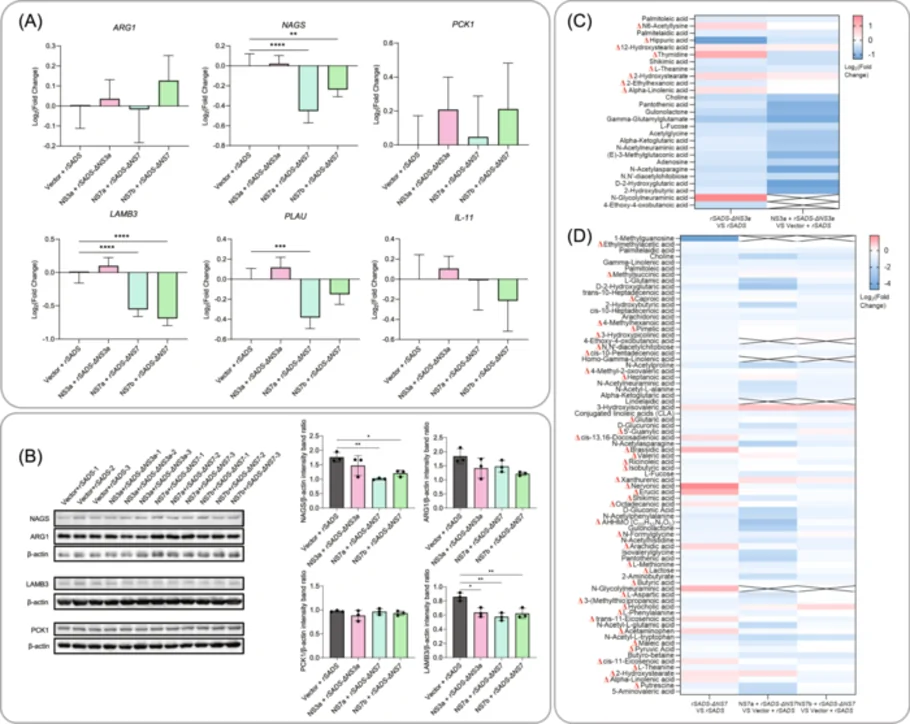
Confirmation of mRNA transcription, protein expression levels, and metabolite levels after compensatory overexpression of proteins in mutated viruses. (A) RT‐qPCR confirmation of mRNA levels of DEGs *ARG1*, *NAGS*, *PCK1*, *LAMB3*, *PLAU*, and *IL‐11* post‐compensatory overexpression. Gene expression levels were calculated using the 2^−ΔΔCt^ method, Vector + *rSADS* group was used as a control group, and *β‐actin* as an endogenous reference. (B) Western blot confirmation of protein expression levels of DEGs ARG1, NAGS, PCK1, and LAMB3 post‐compensatory overexpression. All experiments were repeated at least three times. Heatmaps of relative expression levels of metabolites before and after (C) NS3a or (D) NS7a/NS7b compensatory overexpression. Red shading indicates upregulation, blue shading indicates downregulation, and red triangles indicate significant changes in relative expression levels of metabolites before and after compensation. AHHMO (C_10_H_15_N_3_O_5_) is a custom abbreviation for 4‐amino‐1‐[4‐hydroxy‐5‐(hydroxymethyl)‐3‐methoxy‐2‐oxolanyl]‐2‐pyrimidinone.

The results further showed that overexpression of the respective accessory proteins reversed the differences in the original mRNA transcription levels of *ARG1*, *NAGS*, *PCK1*, *LAMB3*, *PLAU*, and *IL‐11* (Figure 6A). Additionally, the protein expression differences for PCK1 were also eliminated (Figure 6B). Notably, NAGS showed a significant decrease in protein expression following overexpression of NS7a and NS7b, whereas LAMB3 showed decreased protein levels following overexpression of NS3a, NS7a, and NS7b, suggesting that accessory proteins NS7a and NS7b impact the protein expression levels of NAGS and LAMB3, while NS3a specifically affects the LAMB3 protein expression level (Figure 6B).

The metabolomic analysis results showed that, after overexpression of NS3a, the original expression level differences in metabolites such as N6‐acetyllysine, hippuric acid, thymidine, 2‐ethylhexanoic acid, and alpha‐linolenic acid were significantly reversed or even inverted (Figure 6C). After overexpression of NS7a, differences in the original expression levels of 26 metabolites, including ethylmethylacetic acid, methylsuccinic acid, caproic acid, and pimelic acid, were significantly reversed or even inverted, and after overexpression of NS7b, the original expression level differences of 33 metabolites, including ethylmethylacetic acid, methylsuccinic acid, 3‐hydroxypicolinic acid, and 5′‐guanylic acid, were also significantly reversed or inverted, with 18 metabolites significantly impacted by the overexpression of both NS7a and NS7b (Figure 6D).

Overall, overexpressing the compensatory viral accessory proteins eliminated or even reversed the differences in mRNA transcription, protein expression levels, and some metabolite expression levels that were observed between cells infected with the accessory protein‐deficient viruses (*rSADS‐ΔNS3a* and *rSADS‐ΔNS7*) and the parental virus, *rSADS*. NS3a significantly affected the transcription levels of the metabolism‐related genes *ARG1*, *NAGS*, and *PCK1*, and the cytokine gene *IL‐11*; the protein expression levels of PCK1 and LAMB3; and the expression levels of several metabolites, such as N6‐acetyllysine, hippuric acid, thymidine, 2‐ethylhexanoic acid, and alpha‐linolenic acid. NS7a/b significantly affected the transcription levels of the *ARG1*, *NAGS*, and *PCK1* genes, the NF‐κB signaling pathway‐related gene *PLAU*, the ECM receptor interaction‐related gene *LAMB3*, and the *IL‐11* gene; the protein expression levels of NAGS, PCK1, and LAMB3; and 41 metabolites, including ethylmethylacetic acid, methylsuccinic acid, caproic acid, and pimelic acid.

### Reduced pathogenicity of *SADS‐CoV* accessory protein mutant strains in piglets

**FIGURE 7:**
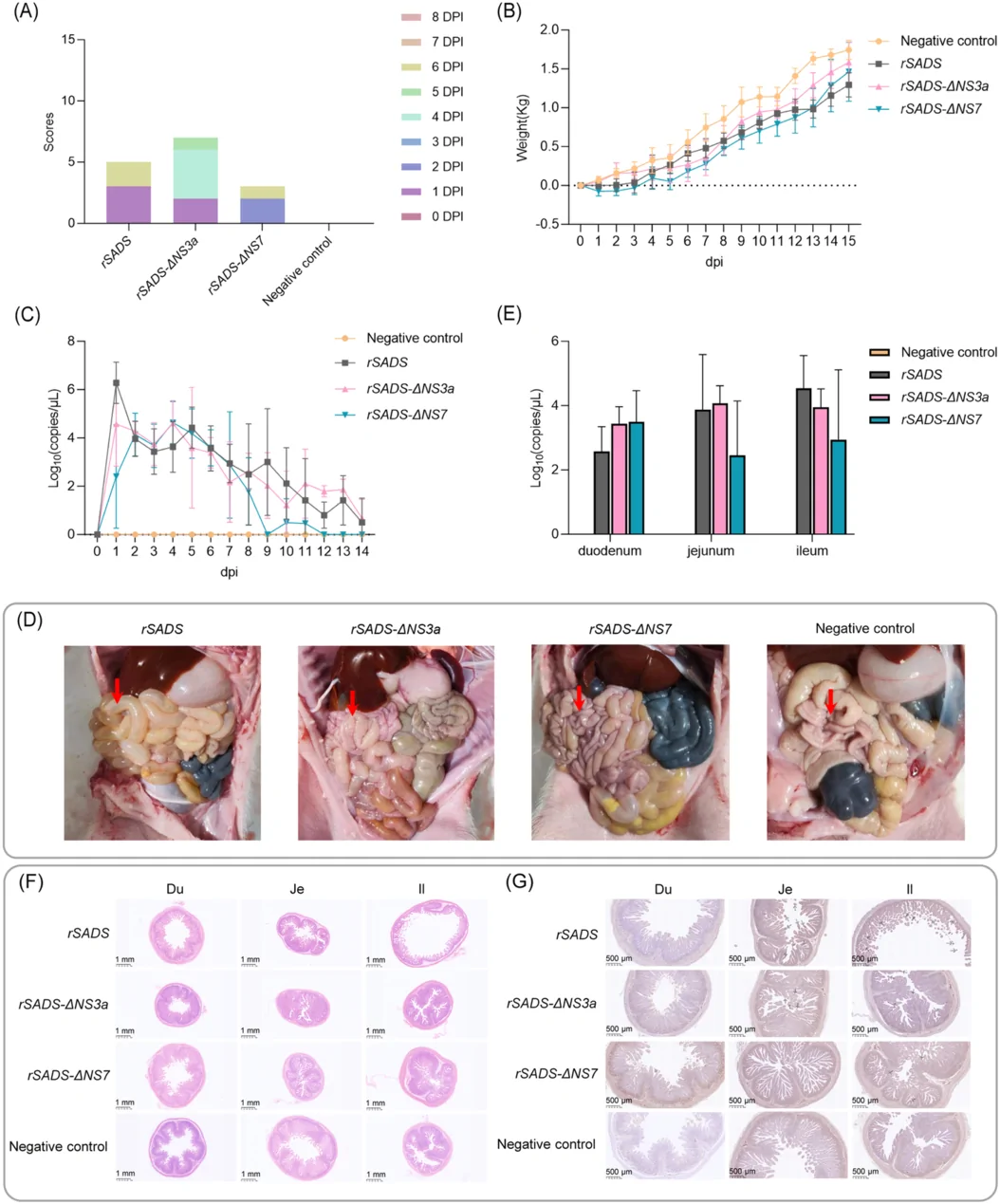
Comparison of clinical and autopsy conditions of piglets infected with the accessory protein deletion mutant viruses and the backbone virus. (A) Cumulative clinical diarrhea scores for piglets after oral ingestion of 10 mL of 10^6.8^TCID_50_/mL *rSADS*, *rSADS‐ΔNS3a*, or *rSADS‐ΔNS7*, or 10 mL DMEM. The severity of diarrhea in piglets was scored as follows: 0 points, normal without diarrhea; 1 point, pasty feces; 2 points, watery feces; 3 points, watery diarrhea/death. (B) Daily cumulative weight gain of piglets postinfection. dpi is the abbreviation for days post infection. (C) Daily quantification of viral RNA in rectal swabs from infected piglets. (D) Intestinal pathological changes of piglets on the 4th day postinfection. Red arrows denote ileal regions in necropsied piglets, with the *rSADS*‐infected group exhibiting marked intestinal wall thinning compared to the other groups. (E) RT‐qPCR detection of *SADS‐CoV* RNA copy numbers in duodenal, jejunal, and ileal tissues of piglets on the 4th day postinfection. (F) H&E staining and (G) IHC analysis targeting the *SADS‐CoV* N protein using histopathological sections of piglet small intestines. Du, Je, and Il are abbreviations for duodenum, jejunum, and ileum, respectively. Arrows indicate the location of intense DAB staining.

To examine the effects of disrupting the NS3a and NS7a/b proteins on pathogenesis using our recombinant viruses, we infected 5‐day‐old piglets. However, neither the mutant viruses (*rSADS‐ΔNS3a* and *rSADS‐ΔNS7*) nor the parental virus (*rSADS*) induced widespread diarrhea. Only some piglets exhibited mild‐to‐moderate diarrhea symptoms, which typically resolved quickly. Consequently, the clinical diarrhea symptom scores were generally low across all groups (Figure 7A), making it challenging to discern differences in virulence among the virus strains based solely on clinical symptoms.

Therefore, we examined changes in weight, viral shedding, intestinal viral load, necropsy status, and histopathological changes to assess differences in virulence. Weight changes showed no significant differences between groups, with only slight variations in the rate of weight gain at different times, suggesting that the absence of accessory proteins does not substantially impact growth of the piglets (Figure 7B).

Daily fecal swabs were collected for viral RNA extraction to enable absolute fluorescence quantification of viral RNA levels. Based on the RNA copy numbers, viral shedding peaked within the first 1‐4 days postinfection, with *rSADS‐ΔNS3a* showing similar shedding patterns to *rSADS*, while *rSADS‐ΔNS7* exhibited significantly reduced viral shedding and was nearly undetectable by day 9 (Figure 7C).

Necropsies performed on day 4 postinfection on three randomly selected piglets from each group showed that, while the control group had thick and intact intestinal walls, the *rSADS*‐infected piglets exhibited noticeable thinning and transparency, indicating severe pathology. In contrast, piglets infected with *rSADS‐ΔNS3a* and *rSADS‐ΔNS7* showed milder changes, suggesting reduced intestinal damage from the viruses lacking the accessory proteins (Figure 7D).

Tissue samples from the duodenum, jejunum, and ileum were also processed for viral RNA quantification. While no *SADS‐CoV* RNA was detected in the control group, the viral RNA was present in all three intestinal sections of the infected groups. However, the viral load was lower in the *rSADS‐ΔNS7* group compared to the *rSADS* and *rSADS‐ΔNS3a* groups, which did not differ significantly from each other (Figure 7E).

Histopathological examination of duodenum, jejunum, and ileum tissue sections from piglets stained with hematoxylin and eosin (H&E) revealed varying degrees of villous atrophy and damage in the small intestine in the challenge groups compared to the negative control group (Figure 7F). Specifically, the *rSADS* challenge group exhibited severe villous atrophy in the ileum, while the *rSADS‐ΔNS3a* and *rSADS‐ΔNS7* challenge groups displayed only mild villous atrophy in the small intestine. These findings suggest that the viruses lacking accessory proteins induce less severe intestinal damage in piglets compared to the parental virus.

Subsequent immunohistochemistry (IHC) analysis detected *SADS‐CoV* antigens in the villous epithelial cells of the duodenum, jejunum, and ileum in the challenge groups to varying extents (Figure 7G). In the *rSADS* challenge group, strong DAB (3,3′‐diaminobenzidine) staining was observed in the jejunum and ileum, with only minimal staining in the duodenum, indicating a high concentration of *SADS‐CoV* antigens in the more severely affected jejunum and ileum. In contrast, the *rSADS‐ΔNS3a* challenge group showed only moderate antigen staining in the ileum and jejunum and minimal staining in the duodenum. The *rSADS‐ΔNS7* challenge group exhibited only minimal staining in the ileum, with very limited staining in the duodenum and jejunum. These results indicate that the viruses with deletions of accessory protein genes caused less severe intestinal damage in piglets compared to the parental virus, with the deletion of the *NS7a/b* genes resulting in a more pronounced reduction in pathogenicity.

In conclusion, the deletion of *NS3a* and *NS7a/b* reduced the virulence of *SADS‐CoV*, with the *NS7a/b* deletion showing a more pronounced effect as evidenced by less extensive viral shedding, lower viral loads in tissues, and milder histopathological changes compared to the parental strain. These findings suggest that the accessory proteins NS3a and NS7a/b play significant roles in the pathogenicity of *SADS‐CoV*.

## 3 DISCUSSION

This study reveals through multi‐omics analyses the critical roles of *SADS‐CoV* accessory proteins NS3a and NS7a/b in modulating host metabolic and immune responses. Existing studies have shown that viral infection reprograms the metabolic networks of carbohydrates, lipids, and amino acids, creating a microenvironment conducive to viral replication while simultaneously suppressing host immune responses, thereby directly contributing to pathogenicity [26]. Other researchers also observed metabolic changes in IPI‐FX porcine intestinal epithelial cells after *SADS‐CoV* infection, reporting that *SADS‐CoV* infection disrupts amino acid metabolism, the tricarboxylic acid (TCA) cycle, and the iron death process in the IPI‐FX cells, with significant impacts on lipid metabolism [27]. Our research has found that the deletion of *NS3a* and *NS7a/b* significantly disrupted the expression of urea cycle enzymes, including NAGS and ARG1, as well as the gluconeogenesis‐related enzyme PCK1. NAGS, a key urea cycle enzyme, sustains cellular homeostasis by preventing ammonia accumulation through urea cycle regulation. Its downregulation disrupts this process, potentially leading to hyperammonemia [28]. ARG1, responsible for converting arginine to urea and ornithine, modulates arginine homeostasis. ARG1 deficiency causes pathological arginine accumulation, which induces immunosuppression, triggers oxidative stress and disrupts metabolic balance [29, 30, 31]. Concurrently, PCK1, the gluconeogenic rate‐limiting enzyme, drives hyperglycemia and immunosuppressive microenvironments when overexpressed, facilitating viral propagation and disease progression [32, 33]. Notably, *NS3a* and *NS7a/b* deletions may mitigate these adverse effects through coordinated upregulation of *NAGS*/*ARG1* and downregulation of *PCK1*, restoring metabolic‐immune equilibrium. These observations suggest that NS3a and NS7a/b may hijack host amino acid and carbohydrate metabolism to modulate viral pathogenicity, consistent with our prior findings in piglets infected with an attenuated *SADS‐CoV* strain deficient in *NS7a/b* [21].

In addition to metabolic reprogramming, *NS3a* and *NS7a/b* deletion significantly impacted the expression of *LAMB3*. LAMB3, a core component of laminins, mediates ECM assembly and stabilization. Its downregulation disrupts ECM integrity, impairs cell migration/tissue repair, and elevates susceptibility to pathogen invasion [34, 35]. Intriguingly, *NS7a/b*‐deficient viral mutants induced elevated *LAMB3* expression in infected cells, which correlated with attenuated intestinal villous damage in piglets‐potentially through restricting pathogen colonization. Notably, *IL‐11* exhibited parallel expression trends with *LAMB3* across infection groups, implying coregulation by viral accessory proteins. As a pleiotropic cytokine, IL‐11 deficiency compromises tissue regeneration and anti‐inflammatory responses, aggravating inflammatory tissue destruction [36, 37]. Thus, *NS3a/NS7a/b* deletions may ameliorate infection‐induced tissue damage via *IL‐11* upregulation, enhancing repair mechanisms while suppressing inflammatory cascades. Recent studies have demonstrated that NLRP1 exerts an anti‐*PDCoV* effect by mediating IL‐11‐induced inhibition of ERK signaling pathway phosphorylation [38]. Based on this finding, we hypothesize that the accessory proteins of *SADS‐CoV* may similarly modulate host responses through this pathway. Although direct mechanistic evidence linking *IL‐11* and *LAMB3* remains limited, their concomitant upregulation in infections of accessory protein‐deficient mutants is closely associated with both the mitigation of virus‐induced damage and the enhancement of host antiviral defense. This observation suggests that *SADS‐CoV* accessory proteins may coordinately modulate host metabolic and immune pathways during pathogenesis, imply a functional interplay between metabolic dysregulation and immunopathogenesis. Future studies will employ LC‐MS/MS and Co‐IP to map these interactions and validate the specific correlation between them.

Based on the KEGG enrichment analysis of the gene expression profiles, we conducted a targeted metabolomics of biomarkers associated with the relevant metabolic pathways, examining the effects of infection with *rSADS‐ΔNS3a* or *rSADS‐ΔNS7* versus control infections with *rSADS* in IPI‐2I cells. As expected, various differential metabolites potentially affected by the accessory proteins NS3a and NS7a/b were identified, and these differential metabolites were mainly concentrated in the pathways for lipid metabolism, amino acid metabolism, and carbohydrate metabolism, consistent with the transcriptomic analysis results and further confirming that the accessory proteins NS3a and NS7a/b impact host lipid, amino acid, and carbohydrate metabolism. The multi‐omics correlation analysis confirmed that *NS7a/b* deletion predominantly perturbed arginine biosynthesis, marked by dysregulated levels of α‐ketoglutarate (α‐KG) and associated metabolites, including l‐aspartic acid, N‐acetyl‐l‐glutamic acid, and l‐glutamic acid (Figure S5). α‐KG, a central TCA cycle intermediate, integrates carbon‐nitrogen metabolism and modulates innate immunity, antioxidant defenses, and energy production [39, 40, 41]. Mechanistically, *SADS‐CoV* accessory proteins NS7a/b may elevate α‐KG levels via glutamine metabolic activation or TCA cycle potentiation [42, 43]. This α‐KG accumulation drives viral pathogenicity by rewiring host metabolism for replication, suppressing immune surveillance, and disrupting redox homeostasis. To rigorously test this hypothesis, Co‐IP experiments can be conducted to validate and further elucidate the precise mechanistic framework through which the *SADS‐CoV* accessory proteins NS7a and NS7b modulate α‐KG metabolism.

In addition to exploring the roles of viral accessory proteins in passaged cell lines in vitro, investigating the effects of deletion strains in animals is an important way to determine whether viral genes are related to virulence. Therefore, we used piglet infections to evaluate changes in viral virulence resulting from the deletion of the accessory protein genes in the porcine pathogen *SADS‐CoV*. Compared to the wild‐type virus, our *NS7a/b* deletion virus caused less virus excretion and lower tissue viral load, and less severe pathological changes in piglets; while the *NS3a* deletion virus also caused less severe pathological changes, the excretion and tissue viral load were not significantly different from those of the wild‐type virus infection group. Therefore, although the absence of either *NS3a* or *NS7a/b* reduced viral virulence, loss of *NS7a/b* led to a greater reduction in virulence, which is consistent with our in vitro results showing a more pronounced effect of *NS7a/b* deletion on fatty acid metabolism, amino acid metabolism, and carbohydrate metabolism processes in this study and corroborates the results of our previous study on piglet infections with an attenuated natural *SADS‐CoV* mutant of *NS7a/b* [44]. It is noteworthy that, unlike earlier studies, piglets infected with *SADS‐CoV* in this study did not exhibit severe clinical symptoms of diarrhea. Instead, they displayed mild‐to‐moderate diarrhea and generally recovered quickly. This observation aligns with the findings reported by Yang et al. in 2020, where piglets challenged with medium‐to‐high doses of purified *SADS‐CoV* also showed mild clinical symptoms, though specific experimental data related to viral dose or clinical symptoms were not provided in their publication [45]. Considering the variability in findings regarding the pathogenicity of *SADS‐CoV* in piglets, it is plausible that multiple factors influence the outcomes of *SADS‐CoV* infection. Further research is required to determine whether the genetic background, weight, and breeding conditions of piglets affect the pathogenicity of *SADS‐CoV*.

Compared to the deletion of the *NS7a/b* genes, why does the deletion of the accessory protein gene *NS3a* have a milder impact on the host? These differences may be due to different structural and functional properties of NS3a and NS7a/b, although further investigation of these properties is needed. NS7a/b likely evolutionarily acquired multifunctional regulatory capacity over host metabolism and immunity. Their deletion triggers broad metabolic reprogramming (lipid, amino acid, and carbohydrate metabolism) and alleviated immunosuppression, severely compromising viral fitness. In contrast, NS3a appears functionally specialized in modulating localized inflammatory responses, with its deletion exerting minimal impact on overall pathogenicity. However, we also hypothesize that the differences in impact may be due to the combined effects of NS7a and NS7b. NS7a/b refers to two distinct accessory proteins, NS7a and NS7b, but their open reading frames largely overlap in the viral genome, making it impossible to individually delete the *NS7a* or *NS7b* gene in the viral genome. However, when we separately complemented the *NS7* deletion mutant with NS7a or NS7b in this study, we observed differences in the host‐related gene expression and metabolite production levels influenced by these two proteins. Thus, we speculate that the more pronounced impact of *NS7a/b* deletion compared to *NS3a* deletion might be attributed to the combined effects of NS7a and NS7b. Additionally, attempts to delete the entire *NS7a* and *NS7b* gene regions resulted in failure to rescue the virus in our initial experiments. This outcome is likely attributable to the extensive deletions at the 3′ end of the viral genome, which may have compromised the genomic stability. To address this issue, future investigations could focus on employing single nucleotide mutations to disrupt the start codons of *NS7a* and *NS7b* separately. This approach would allow for the selective inactivation of each gene, thereby facilitating a more precise analysis of their individual roles without jeopardizing the overall integrity of the viral genome.

## 4 CONCLUSION

This study has elucidated the role of three *SADS‐CoV* accessory proteins in influencing viral pathogenicity and preliminarily explored the host molecular targets and pathways affected by the NS3a, NS7a, and NS7b proteins of *SADS‐CoV*. Our findings suggest that *SADS‐CoV* accessory proteins may modulate viral pathogenicity by affecting host metabolic pathways and immunity. These insights provide a foundation for future strategies aimed at mitigating the impact of *SADS‐CoV* infection in piglets, either by targeting specific accessory proteins or their associated molecular pathways, and offer valuable guidance for developing coronavirus vaccines by deleting the relevant accessory protein genes.

## 5 METHODS

### Ethics statement

This study complied with the National Standards for Laboratory Animals of the People's Republic of China (GB149258‐2010) and was approved by the Animal Research Committees of South China Agricultural University (Ethics No. 2024f088). Pigs used for the study were handled in accordance with good animal practices required by the Animal Ethics Procedures and Guidelines of the People's Republic of China.

### Cells and viruses

The *SADS‐CoV/GDWT/2017* strain (GenBank accession no. MG557844) was isolated in Guangzhou, China, in April 2017 [3]. African green monkey kidney cells (Vero) were cultured in Dulbecco's modified Eagle's medium (DMEM) with 10% (vol/vol) fetal bovine serum and 1% (vol/vol) penicillin‐streptomycin, and used to rescue *SADS‐CoV/GDWT/2017*‐derived recombinants and to determine their titer. Porcine ileal epithelial cells (IPI‐2I) were cultured under identical conditions and used for one‐step growth curves, transcriptomics, metabolomics, RT‐qPCR, and western blot analyses of the *SADS‐CoV/GDWT/2017*‐derived recombinants.

### *Escherichia coli* strains and plasmids

pBeloBAC11‐cm‐CI‐SADS‐CoV‐WT‐F1‐F14‐HDVrz‐SV40polyA is the full‐length infectious clone of *SADS‐CoV* obtained by generation of *SADS‐CoV/GDWT/2017* genomic cDNA. *E. coli* GB08‐red‐gyrA462 was used for the deletion of the viral accessory protein genes from pBeloBAC11‐cm‐CI‐SADS‐CoV‐WT‐F1‐F14‐HDVrz‐SV40polyA, and *E. coli* GB2005 was used for transformation with plasmids constructed by Gibson assembly. p15A‐amp‐ccdB was used as a template to amplify the *amp‐ccdB* resistance gene. p15A‐cm‐CAGGS‐HA‐BGH‐polyA was used to construct eukaryotic overexpression plasmids. The plasmids (p15A‐amp‐ccdB, p15A‐cm‐CAGGS‐HA‐BGH‐polyA) and bacterial strains (*E. coli* GB08‐red‐gyrA462, *E. coli* GB2005) for Redα/β recombination and Gibson assembly can be freely requested from our laboratory (Shandong University) for academic research.

### Knockout of the accessory protein gene *NS3a* and *NS7a/b*

DNA fragments ΔNS3a(all)‐ampccdB, ΔNS7a/b‐58bp‐ampccdB, and ΔNS7a/b‐58bp (a 58 bp deletion spanning the TRS of NS7a/b sgRNA) were obtained through PCR amplification using oligonucleotides that contained homologous arms with AscI restriction sites (Table S1). After gel electrophoresis, the linear fragments were recovered from the gel. Using the infectious clone pBeloBAC11‐cm‐CI‐SADS‐CoV‐WT‐F1‐F14‐HDVrz‐SV40polyA, the *NS3a* and *NS7a/b* genes were deleted by Redα/β recombination using, respectively, the ΔNS3a(all)‐ampccdB or ΔNS7a/b‐58bp‐ampccdB fragment in *E. coli* GB08‐red‐gyrA462. The recombinants were selected on LB plates containing 100 μg/mL ampicillin and 15 μg/mL chloramphenicol. After confirmation by restriction enzyme analysis, the *amp‐ccdB* segments of the correct clones were removed by AscI (NEB, R0558L) digestion. The resulting linear fragments were then combined with or without the fragment ΔNS7a/b‐58bp using the Gibson Assembly® Master Mix (2×) (NEB, E2611S) and transformed into *E. coli* GB2005 to obtain the recombinant plasmids pBeloBAC11‐cm‐CI‐SADS‐CoV‐WT‐F1‐F14‐HDVrz‐SV40polyA‐ΔNS3a and pBeloBAC11‐cm‐CI‐SADS‐CoV‐WT‐F1‐F14‐HDVrz‐SV40polyA‐ΔNS7. The recombinants were selected on LB plates containing 15 μg/mL chloramphenicol, and the correct recombinants were identified by PstI (NEB, R3140L) restriction enzyme analysis and PCR analysis (Table S2). Sequencing of recombinant plasmids was conducted by Sangon Biotech Co., Ltd.

The infectious clones and the helper plasmid pCI‐amp‐SADS‐CoV‐N were transfected into Vero cells using Lipofectamine® 3000 (Thermo Fisher Scientific, L3000015) to rescue the viruses *rSADS‐CoV‐ΔNS3a* and *rSADS‐CoV‐ΔNS7*.

### One‐step growth curves of rescued viruses

IPI‐2I cells were infected at an MOI of 0.1, and after 1 h, the medium was replaced with DMEM containing 5 μg/mL trypsin. Supernatants were collected at various time points (0, 3, 6, 9, and 12 h postinfection), and used to infect Vero cells in 96‐well plates to determine viral TCID_50_ (50% tissue culture infectious dose) at each time point. Additionally, 200 μL of supernatant from each time point was used to extract viral nucleic acids using the AxyPrep Body Fluid Viral DNA/RNA Miniprep Kit (Axygen, AP‐MN‐BF‐VNA‐250), followed by quantification of SADS‐CoV copy number at different time points by RT‐qPCR of the viral *N* gene using the HiScript II U+ One Step RT‐qPCR Probe Kit (Vazyme, Q223‐01) (Table S3).

### Transcriptomic analysis

IPI‐2I cells were infected with 0.1 MOI of *rSADS*, *rSADS‐ΔNS3a*, or *rSADS‐ΔNS7* per well (20 wells per virus) in 2 mL of DMEM containing 5 μg/mL trypsin. After 1 h at 37°C, the medium was replaced with fresh DMEM containing 5 μg/mL trypsin. At 3, 6, 9, and 12 h postinfection, samples from 5 wells per time point were collected, lysed with 1 mL of Trizol, and stored at −80°C. Samples were sent to Lc‐Bio Technologies Co., Ltd. for transcriptome sequencing.

The total RNA quantity and purity were assessed using the Bioanalyzer 2100 and RNA 6000 Nano LabChip Kit (Agilent, 5067‐1511); high‐quality RNA samples with an RNA integrity number (RIN) > 7.0 were used to construct the sequencing library. A cDNA library was constructed from the pooled RNA from the IPI‐2I cell samples and then sequenced with the Illumina Novaseq™ 6000 (Illumina) sequencing platform; using the Illumina paired‐end RNA‐seq approach, we generated a total of million 2 × 150 bp paired‐end reads for the transcriptome. To remove low‐quality raw reads and obtain only high‐quality clean reads, the reads were further filtered by Cutadapt (https://cutadapt.readthedocs.io/en/stable/, version: cutadapt‐1.9). Then, we aligned reads of all samples to the *sus scrofa* reference genome using HISAT2 (https://daehwankimlab.github.io/hisat2/, version: hisat2‐2.2.1) package, which initially remove a portion of the reads based on quality information accompanying each read and then maps the reads to the reference genome. HISAT2 allows multiple alignments per read (up to 20 by default) and a maximum of two mismatch when mapping the reads to the reference. HISAT2 build a database of potential splice junctions and confirms these by comparing the previously unmapped reads against the database of putative junctions. The mapped reads of each sample were assembled using StringTie (http://ccb.jhu.edu/software/stringtie/, version: stringtie‐2.1.6) with default parameters. Then, all transcriptomes from all samples were merged to reconstruct a comprehensive transcriptome using gffcompare software (http://ccb.jhu.edu/software/stringtie/gffcompare.shtml, version: gffcompare‐0.9.8). After the final transcriptome was generated, StringTie and ballgown (http://www.bioconductor.org/packages/release/bioc/html/ballgown.html) were used to estimate the expression levels of all transcripts and perform expression abundance for mRNAs by calculating FPKM (fragment per kilobase of transcript per million mapped reads) value.

Genes differential expression analysis was performed by DESeq. 2 version 1.22.2 software between two different groups (and by edgeR version 3.22.5 between two samples). The threshold criteria for selecting DEGs were set as a fold change (FC) of ≥2 (i.e., an absolute value of log_2_FC ≥ 1) and a *q* value < 0.05 (where the *q* value is the adjusted *p‐*value) (|log_2_FC| ≥ 1 & *q* < 0.05). DEGs were then subjected to enrichment analysis of GO functions in the Gene Ontology database (http://www.geneontology.org/) and KEGG pathways in the Kyoto Encyclopedia of Genes and Genomes database (https://www.kegg.jp/kegg/). The calculating formula of *p‐*value is:

p=1−∑i=0m−1MiN−Mn−iNn,

*N* is the number of all genes with GO/KEGG annotation, *n* is the number of DEGs in *N*, *M* is the number of all genes annotated to the certain GO/pathways, and *m* is the number of DEGs in *M*. GO terms/Pathways meeting this condition with *p* < 0.05 were defined as significantly enriched GO terms/pathways in DEGs.

### Metabolomic analysis

IPI‐2I cells were infected at an MOI of 0.1 and harvested at 12 h postinfection, snap‐frozen in liquid nitrogen for 15 min and subsequently stored at −80°C. Samples were sent to Novogene Co., Ltd. for targeted metabolomic analysis using the N500 platform.

All of the standards were obtained from ZZ Standards Co., Ltd. Shanghai Aladdin Biochemical Technology Co., Ltd., and Sigma‐Aldrich Co., Ltd. Methanol (Optima LC‐MS), acetonitrile (Optima LC‐MS), formate (Optima LC‐MS), isopropanol (Optima LC‐MS) were purchased from Thermo‐Fisher Scientific (FairLawn); 3‐nitrophenylhydrazine (3‐NPH) (Optima LC‐MS) was purchased from Sigma‐Aldrich; and 1‐(3‐dimethylaminopropyl)‐3‐ethylcarbodiimide (EDC) (Optima LC‐MS) and pyridine (Optima LC‐MS) were purchased from Shanghai Aladdin Biochemical Technology Co., Ltd. Ultrapure water was purchased from Millipore. Stock solutions of individual metabolites were mixed and prepared in metabolite‐free matrix to obtain a series of metabolite calibrators. Certain concentrations of isotope standard were compounded and mixed as internal standards. All stock solutions and working solutions were stored in a −20°C freezer.

The samples were freezed‐dried and added to water (100 μL) by well vortexing. Then homogenized with 300 μL of methanol (80%) and stewing 10 min. Then centrifuged at 15,000 rpm for 15 min (4°C) to remove the protein. The supernatant (50 μL) was added to derivatization reagent (150 μL) and derivatized at 40°C for 40 min. After that, added to water by well vortexing as the diluted sample. The supernatant (90 μL) was homogenized with 10 μL mixed internal standard solution. Finally, injected into the LC‐MS/MS system for analysis. An ultrahigh performance liquid chromatography coupled to tandem mass spectrometry (UHPLC‐MS/MS) system (ExionLC™ AD UHPLC‐QTRAP 6500+, AB SCIEX Corp.) was used to quantitate metabolites at Novogene Co., Ltd. Separation was performed on a Waters HSS T3 column (2.1 × 150 mm), which was maintained at 40°C. The mobile phase, consisting of 0.1% formic acid in water (solvent A) and acetonitrile/isopropanol (1:1) (solvent B), was delivered at a flow rate of 0.30 mL/min. The solvent gradient was set as follows: 5% B, 1 min; 5%–40% B, 7 min; 40%–95% B, 25 min; 95%–5% B, 27.1 min; and 5% B, 30 min. The mass spectrometer was operated in positive/negative multiple reaction mode (MRM) mode. Parameters were as follows: ionspray voltage (4500 V/−4500 V), sheath gas (35 psi), ion source temperature (550°C), auxiliary gas (50 psi), and collision gas (55 psi). LC‐MS was used to detect the concentration series of standard solution. The ratio of the concentration of standard to internal standard as abscissa, and the ratio of peak area of standard to internal standard as ordinate to investigate the linearity of standard solution. The correlation coefficient (*r*) > 0.99 of each metabolites were the necessary condition. The limit of quantification (LOQ) was determined by the method of signal‐to‐noise ratio (S/N), which is comparing the signal measured by the standard solution concentration with the blank matrix. Generally, when the S/N = 10:1, the corresponding concentration is the LOQ [46, 47]. The selection of differential metabolites was primarily based on three parameters: VIP, FC, and *p*‐value. Variable importance in the projection (VIP) refers to the first principal component in the PLS‐DA model, indicating the contribution of metabolites to the group differentiation [48]. Fold change (FC) is the ratio of the average quantitative value of each metabolite in all biological replicates of the comparison groups. The *p‐*value is calculated using the *T*‐test and indicates the level of significance of the differences [47]. The thresholds were set as VIP > 1.0, FC > 1.2 or FC < 0.833, and *p*‐value < 0.05 [49, 50, 51].

### Total RNA extraction and fluorescent quantitative PCR identification

Total RNA was extracted from infected cells using RNAiso Plus (Takara, D9108A) according to the manufacturer's instructions, and stored at −80°C until use. The extracted RNA was then reverse‐transcribed using the HiScript III 1st Strand cDNA Synthesis Kit (+gDNA wiper) (Vazyme, R312‐02) according to the manufacturer's instructions. The levels of viral RNA and target gene expression in the samples were detected by real‐time fluorescence quantitation PCR using the Applied Biosystems QuantStudio™ 3 Real‐Time PCR Systems (Thermo Fisher Scientific) and primers listed in Table S4. Gene expression levels were calculated using the 2^−ΔΔCt^ method [52], and data were analyzed using the one‐way ANOVA function of GraphPad Prism software (version 10.1.2).

### Western blot analysis

Total Proteins was extracted from infected cells using RIPA buffer containing protease inhibitors according to the manufacturer's instructions, and stored at −80°C until use. Proteins were electrophoresed on a 10% SDS‐PAGE gel. A PVDF membrane of appropriate size was cut and activated in anhydrous ethanol, and then soaked in transfer buffer. Protein transfer was carried out using a universal protein transfer system following standard protocols. After the transfer was complete, the PVDF membrane was carefully removed and blocked in 5% skim milk; the membrane was then washed with Tris‐buffered saline containing Tween 20 (TBST) and incubated overnight at 4°C with polyclonal antibodies for NAGS (Proteintech, 21566‐1‐AP, 1:500); arginase‐1 (Proteintech, 16001‐1‐AP, 1:5000); PCK1 (Proteintech, 16754‐1‐AP, 1:5000); or LAMB3 (Proteintech, 26795‐1‐AP, 1:1000). The membrane was washed with TBST and incubated with the secondary antibody, horseradish peroxidase‐conjugated Affinipure goat anti‐rabbit IgG (H + L) (Proteintech, SA00001‐2, 1:10000), on a shaker at room temperature for 1 h, followed by washing with TBST and exposure using the ECL method. Protein bands were detected, and grayscale values were analyzed using Image J software (version 1.54g).

### Construction of eukaryotic overexpression plasmids

Primers were designed with homologous arms on both ends of each primer sequence. pBeloBAC11‐cm‐CI‐SADS‐CoV‐WT‐F1‐F14‐HDVrz‐SV40polyA was used as the PCR template, and primer pairs Flag‐NS3a‐F/NS3a‐R, Flag‐NS7a‐F/NS7a‐R, and Flag‐NS7b‐F/NS7b‐R were used to amplify the Flag‐NS3a, Flag‐NS7a, and Flag‐NS7b fragments, respectively (Table S1). The Flag fragment was only amplified by primers Flag‐F and Flag‐R (Table S1). The p15A‐cm‐CAGGS‐HA‐BGH‐polyA plasmid was linearized using the restriction enzyme NotI (NEB, R0189L). PCR amplification and enzyme digestion products were electrophoresed on a 1% agarose gel, and the desired bands were cut out and recovered using the E.Z.N.A® Gel Extraction Kit (Omega, D2500‐01). The concentrations were measured, and fragments were assembled in vitro using the Gibson Assembly® Master Mix (2×). The assembly products were transformed into *E. coli* GB2005, which were then plated on LB solid media containing 15 μg/mL chloramphenicol. Single colonies were cultured in LB liquid media containing 10 μg/mL chloramphenicol, and the correct clones were identified by colony PCR and sent to Sangon Biotech Co., Ltd. for sequencing.

### IFA for protein expression identification

Lipofectamine® 3000 Reagent was used to transfect the IPI‐2I cells with 2 µg of overexpression plasmid per well. At 24 h posttransfection, cells were fixed with 4% paraformaldehyde. After removing the fixative, the cells were washed with PBS and permeabilized with Triton X‐100. The permeabilization solution was discarded, followed by blocking with QuickBlock™ immunostaining blocking solution (Beyotime, P0260). Samples were washed with TBST and incubated overnight at 4°C with mouse monoclonal ANTI‐FLAG® M2 antibody (Sigma‐Aldrich, B3111‐0.2MG) diluted 1:1000. After discarding the primary antibody solution, samples were washed with TBST and incubated with Multi‐rAb CoraLite® Plus 488‐Goat Anti‐Mouse Recombinant Secondary Antibody (H + L) (Proteintech, RGAR002), diluted 1:500, at room temperature for 1 h. After discarding the secondary antibody solution, samples were washed with TBST, Beyotime anti‐fading mounting medium containing 4′,6‐diamidino‐2‐phenylindole (DAPI) (Beyotime, P0131‐5mL) was added, and fluorescence was observed under an inverted fluorescence microscope.

### Assessment of functional compensation using the overexpression plasmids

Cells were prepared 1 day in advance, with IPI‐2I cells seeded in six‐well plates (for total RNA and protein extraction) or 100 mm dishes (for metabolite extraction). When the cells reached about 60% confluence the next day, transfection was carried out using Lipofectamine® 3000 Reagent, using 2 µg of overexpression plasmid per well in 6‐well plates, and 10 µg of plasmid per dish in 100 mm dishes. One day later, when the cells reached about 90% confluence, cell culture medium was discarded, and then 2 mL (for wells) or 10 mL (for plates) of DMEM containing 5 μg/mL trypsin was added. The wells/dishes transfected with pCAGGS‐Flag were infected with a 0.1 MOI of *rSADS*, those with pCAGGS‐Flag‐NS3a were infected with a 0.1 MOI of *rSADS‐ΔNS3a*, and those with pCAGGS‐Flag‐NS7a and pCAGGS‐Flag‐NS7b were infected with a 0.1 MOI of *rSADS‐ΔNS7*. At 12 h postinfection, total RNA, protein, or metabolites were collected for analysis as needed.

### Animal experiments

Twenty‐six 5‐day‐old piglets, negative for *SADS‐CoV* antibodies, antigens, and other diarrheal pathogens, were selected. For the *rSADS*, *rSADS‐ΔNS3a*, and *rSADS‐ΔNS7* virus infection groups, seven piglets per group were orally administered 10 mL of 10^6.8^TCID_50_/mL of virus fluid, and the remaining five piglets served as negative controls and were administered 10 mL of DMEM. Postinfection, each piglet's clinical condition was observed and photographed daily, and their weight was measured to calculate and record daily weight gain. The severity of diarrhea in piglets was scored as follows: 0 points, normal without diarrhea; 1 point, pasty feces; 2 points, watery feces; 3 points, watery diarrhea/death.

Daily, anal swabs were collected from each piglet, added to 1 mL of PBS, shaken, then centrifuged at 12,000 rpm for 1 min, and the supernatant was collected. Viral nucleic acids were extracted using the AxyPrep Body Fluid Viral DNA/RNA Miniprep Kit, and the shedding of *SADS‐CoV* was detected using the HiScript II U+ One Step RT‐qPCR Probe Kit.

### Tissue sample collection and viral copy number detection

Four days postinfection, three piglets from each group were randomly selected and euthanized for necropsy. Tissues from the duodenum, jejunum, and ileum were collected. Each tissue sample (0.5 g) was added to 1 mL of PBS and ground. Viral nucleic acids were extracted using the AxyPrep Body Fluid Viral DNA/RNA Miniprep Kit, and *SADS‐CoV* viral copy numbers in each tissue were detected using the HiScript II U+ One Step RT‐qPCR Probe Kit.

### H&E staining for intestinal histopathology

Tissue samples of 1–2 cm in size from the duodenum, jejunum, and ileum of each group were fixed in 4% formaldehyde solution for 48 h, and then embedded in paraffin, sectioned, and subjected to H&E staining. Dehydration was performed stepwise using low‐to‐high concentrations of alcohol, followed by embedding in paraffin and further processing and then preparation of tissue sections using standard procedures. The sections were stained with Harris hematoxylin for 3–5 min, rinsed in tap water for 1–2 min, differentiated in 0.8%–1% hydrochloric acid alcohol, and rinsed again in tap water. Bluing was performed in a diluted lithium carbonate solution, followed by rinsing, and then staining in eosin (alcohol‐soluble) for 1–2 s. Dehydration was performed by immersing the sections in 95% ethanol and absolute ethanol for 1–2 min each. The sections were then cleared in xylene, mounted, and scanned with a brightfield scanner.

### IHC of intestinal tissue

Separate sections of the above paraffin‐embedded samples were processed in synchrony for IHC. Paraffin‐embedded sections were deparaffinized three times with an eco‐friendly deparaffinizing agent for 10 min each and then rehydrated through a graded series of ethanol: absolute, 95%, and 75% for 5 min each. Samples were washed with Tris‐buffered saline (TBS), and then citrate buffer (pH 6.0) was added before further processing by boiling, cooling, and then washing with TBS, before incubation in 3% hydrogen peroxide at room temperature for 30 min to block endogenous peroxidase activity. Samples were then rinsed with TBS, placed in TBST, and then blocked with 10% goat serum at room temperature for 30 min. After discarding the serum, the primary antibody (anti‐*SADS‐CoV* N protein mouse polyclonal antibody), which was diluted 1:900 in 10% goat serum, was added followed by incubation overnight at 4°C. After washing with TBST, goat anti‐mouse secondary antibody was added followed by incubation at 37°C for 45 min, and washing again with TBST. After discarding the TBST, freshly prepared DAB was added, and the reaction was terminated with tap water after an appropriate period of time. Samples were then processed by staining with hematoxylin and bluing solution to color the nuclei and then mounting using standard protocols before scanning with a brightfield scanner.

### Data analysis

All results are presented as the mean ± SD of three completely independent experiments, and Student's *t*‐test was used for comparison between two groups. To make comparisons among more than two groups, all data were first analyzed for conformity to a normal distribution, followed by one‐way ANOVA, and then Tukey's post‐hoc test. *p < 0.05, **p < 0.01, ***p < 0.001, and ****p < 0.0001 were considered statistically significant. All data were analyzed using GraphPad Prism (version 10.1.2) statistical software. The data and statistical graphic project files used are saved in GitHub (https://github.com/Xiaoling-Yan/Multi-omics-of-SADS).

## AUTHOR CONTRIBUTIONS

**Jingyun Ma** and **Jun Fu**: Conceptualization; study design; revision. **Xiaoling Yan**: Experimentation; data analysis; writing—original draft. **Xiaoli Zhang**: Experimentation; data analysis. **Ling Zhou, Xiaoya Zhao, Qianniu Li,** and **Tian Lan**: Supporting experimental implementation. All authors have read the final manuscript and approved it for publication.

## CONFLICT OF INTEREST STATEMENT

The authors declare no conflicts of interest.

## ETHICS STATEMENT

The ethics application (No. 2024f088) was approved by the Laboratory Animal Ethics Committee of South China Agricultural University.

## Supporting information

The online version contains supplementary figures and tables available.

Figure S1 Schematic of the construction of the infectious clones for the *SADS‐CoV* accessory protein gene deletion strains.
Figure S2 Rescue and characterization of *SADS‐CoV* accessory protein gene deletion mutants.
Figure S3 iPath pathway map of the commonly enriched pathways of differential genes and differential metabolites in the comparison group *rSADS‐ΔNS7* vs. *rSADS*.
Figure S4 Validation of transcriptional and protein expression levels of overexpression plasmids post‐transfection in IPI‐2I cells.
Figure S5 Pathview enriched pathway map showing DEGs and differential metabolites in the arginine biosynthesis pathway for the comparison group *rSADS‐ΔNS7* vs. *rSADS*.
Table S1 Primer sequences for amplification of DNA fragments required for construction of *NS3a*/*NS7a/b* deletion recombinant plasmids and eukaryotic expression vectors.
Table S2 PCR validation primer sequences for *SADS‐CoV NS3a* and *NS7a/b* sites.
Table S3 Fluorescence quantitation detection primers and probe sequences for *SADS‐CoV*.
Table S4 Primer sequences for relative fluorescence quantitative PCR.

## Data Availability

All the sequencing data have been deposited in GSA under GSA accession number CRA022998 (transcriptomic sequencing), BioProject accession number PRJCA036150 (https://bigd.big.ac.cn/gsa/browse/CRA022998). The data and statistical graphic project files used are saved in GitHub (https://github.com/Xiaoling-Yan/Multi-omics-of-SADS). Supplementary materials (figures, tables, graphical abstract, slides, videos, Chinese translated version, and update materials) may be found in the online DOI or iMeta Science (http://www.imeta.science/imetaomics/).
