# Supplementary material for: Effects of SADS‐CoV accessory proteins NS3a, NS7a, and NS7b on viral pathogenicity: A multi‐omics investigation
Source: IMetaOmics. 2025 Apr 6;2(3):e70015. doi: 10.1002/imo2.70015 (PMC12806138; doi:10.1002/imo2.70015)
Supplement: Supplementary file 1 — Figure S1 Schematic of the construction of the infectious clones for the SADS‐CoV accessory protein gene deletion strains. Figure S2 Rescue and characterization of SADS‐CoV accessory protein gene deletion mutants. Figure S3 iPath pathway map of the commonly enriched pathways of differential genes and differential metabolites in the comparison group rSADS‐ΔNS7 vs. rSADS. Figure S4 Validation of transcriptional and protein expression levels of overexpression plasmids post‐transfection in IPI‐2I cells. Figure S5 Pathview enriched pathway map showing DEGs and differential metabolites in the arginine biosynthesis pathway for the comparison group rSADS‐ΔNS7 vs. rSADS. Table S1 Primer sequences for amplification of DNA fragments required for construction of NS3a/NS7a/b deletion recombinant plasmids and eukaryotic expression vectors. Table S2 PCR validation primer sequences for SADS‐CoV NS3a and NS7a/b sites. Table S3 Fluorescence quantitation detection primers and probe sequences for SADS‐CoV. Table S4 Primer sequences for relative fluorescence quantitative PCR. [file IMO2-2-e70015-s001.docx]

**Supporting information to：**

**Effects of *SADS-CoV* accessory proteins NS3a, NS7a, and NS7b on viral pathogenicity: a multi-omics investigation**

**Running title:** *SADS-CoV* NS3a/NS7a/b modulate host metabolism and virulence

Xiaoling Yan^1,2^, Xiaoli Zhang^1,2^, Ling Zhou^1,2^, Xiaoya Zhao^1,2^, Qianniu Li^1,2^, Tian Lan^1,2^*, Jun Fu^3^*, Jingyun Ma^1,2^*

1 State Key Laboratory of Swine and Poultry Breeding Industry, Guangzhou 510642, China

2 College of Animal Science, South China Agricultural University, Guangzhou 510642, China

3 Shandong University–Helmholtz Institute of Biotechnology, State Key Laboratory of Microbial Technology, Shandong University, Qingdao 266237, China

* Correspondence:

[lantian2016@scau.edu.cn](mailto:lantian2016@scau.edu.cn) (Tian Lan)

[fujun@sdu.edu.cn](mailto:fujun@sdu.edu.cn) (Jun Fu)

[majy2400@scau.edu.cn](mailto:majy2400@scau.edu.cn) (Jingyun Ma)

***
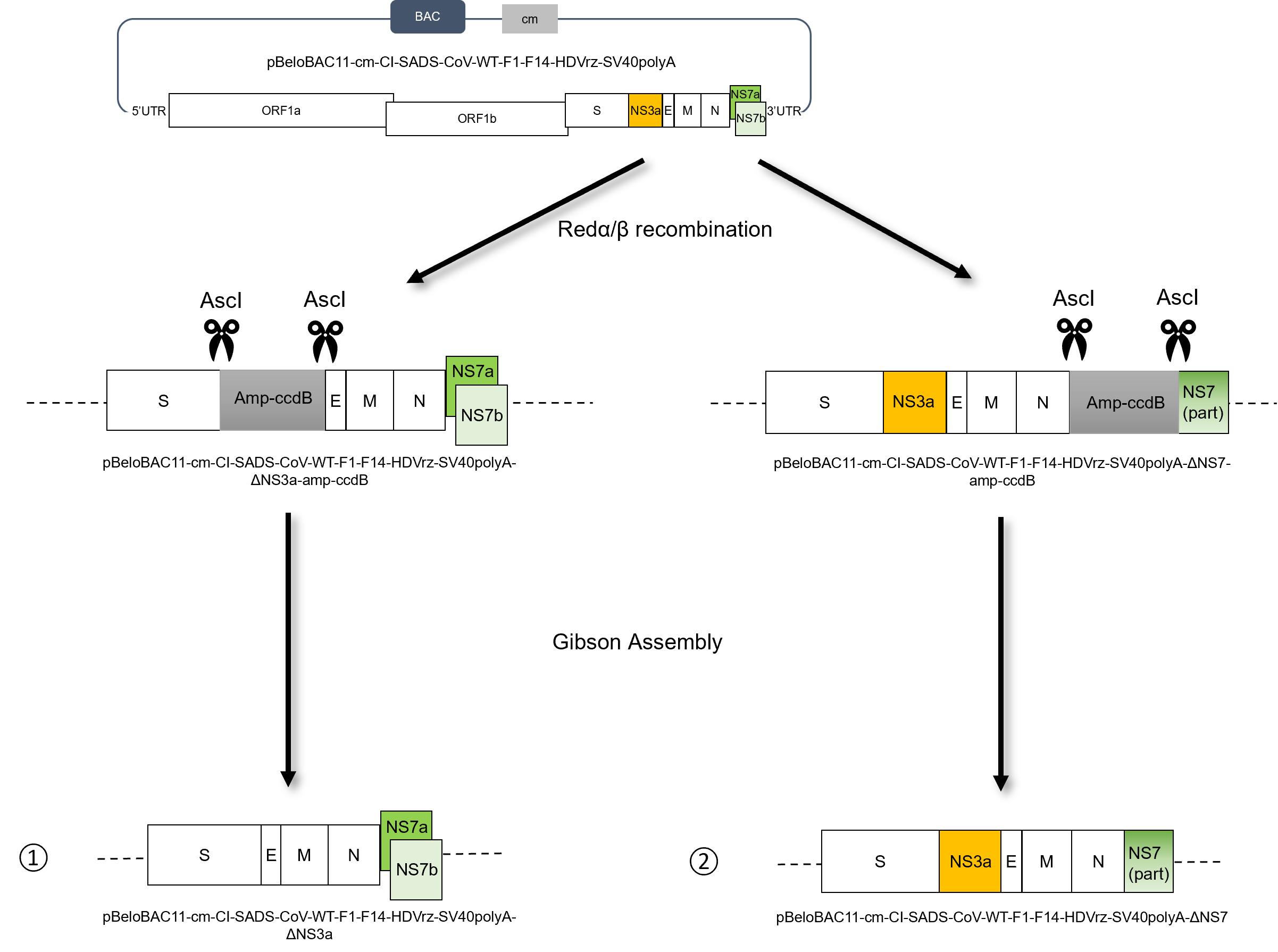
***

**Figure S1** Schematic of the construction of the infectious clones for the *SADS-CoV* accessory protein gene deletion strains. Construction of the infectious clones for the *NS3a* and *NS7a/b* deletion strains was completed using two rounds of recombination. In the first round of recombination, the fragments ΔNS3a(all)-ampccdB and ΔNS7-ampccdB were recombined separately with the plasmid pBeloBAC11-cm-CI-SADS-CoV-WT-F1-F14-HDVrz-SV40polyA by Redα/β recombination. In the second round of recombination, the linearized plasmids that had the *amp-ccdB* segment removed by AscI digestion were self-ligated or assembled with the ΔNS7a/b-58bp fragment (for pBeloBAC11-cm-CI-SADS-CoV-WT-F1-F14-HDVrz-SV40polyA-ΔNS7) through Gibson assembly.


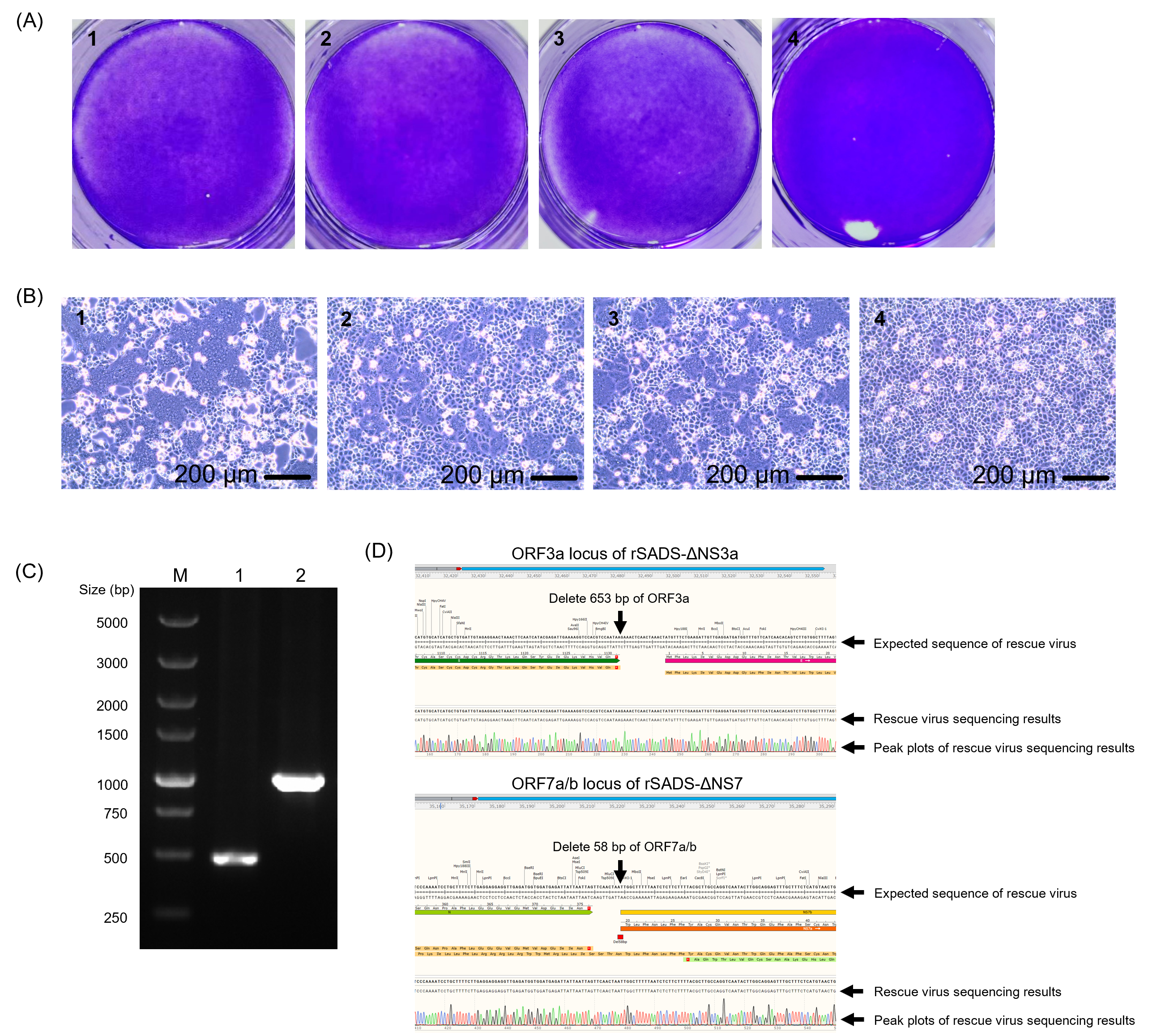


**Figure S2** Rescue and characterization of *SADS-CoV* accessory protein gene deletion mutants. (A, B) Vero cells infected with the rescued viruses *rSADS*, *rSADS-ΔNS3a*, and *rSADS-ΔNS7* and examined by (A) crystal violet staining for visual observation of cytopathic lesions and (B) microscopic observation of cytopathic lesions. Cells were infected with (1) *rSADS*, (2) *rSADS-ΔNS3a*, or (3) *rSADS-ΔNS7*, or were (4) mock-infected. (C) PCR identification of the rescued viruses *rSADS-ΔNS3a* and *rSADS-ΔNS7*; lane 1, ORF3a locus of *rSADS-ΔNS3a*; lane 2, ORF7a/b locus of *rSADS-ΔNS7*; M, marker lane. (D) Sequencing validation results of the ORF3a locus of *rSADS-ΔNS3a* and the ORF7a/b locus of *rSADS-ΔNS7*.

***
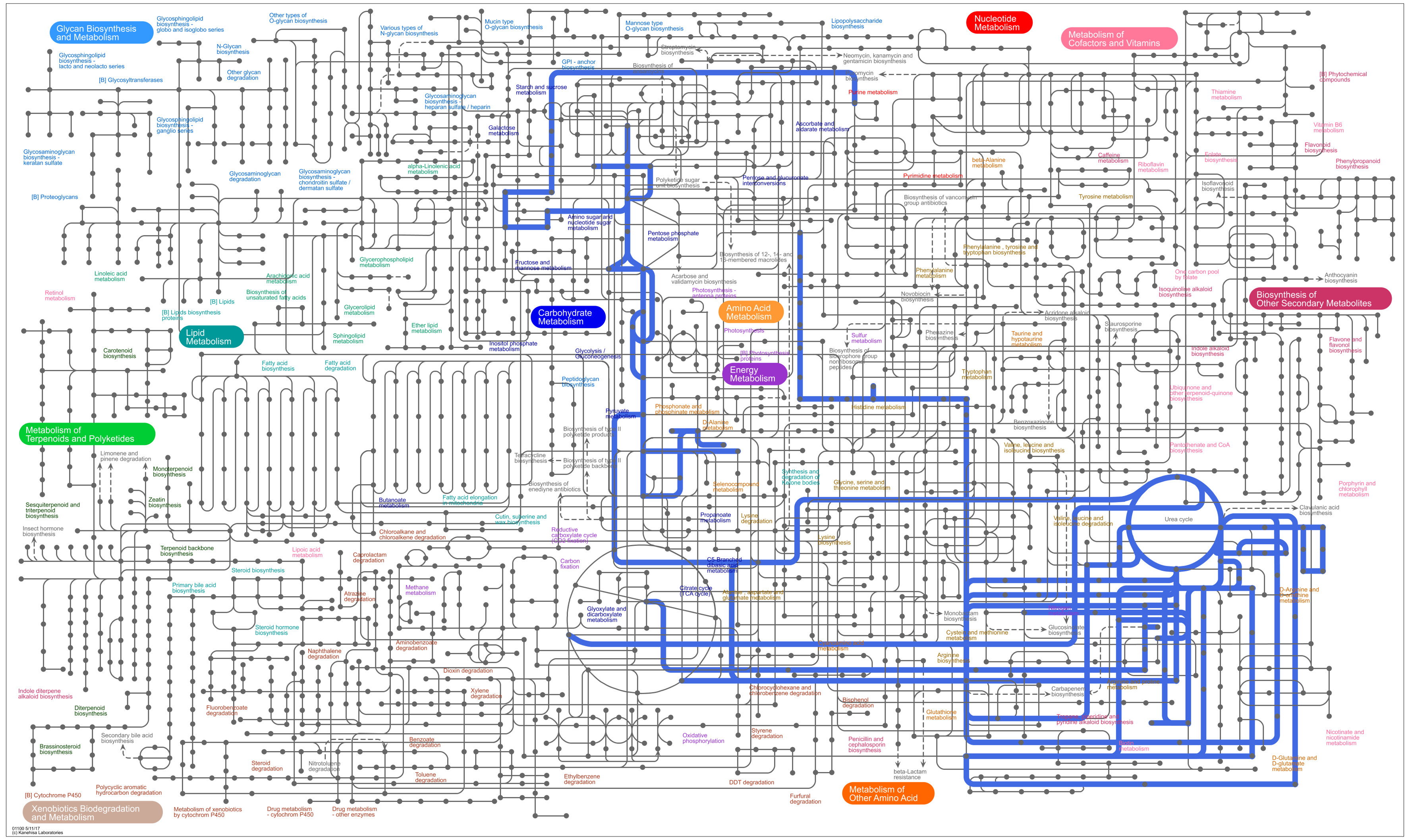
***

**Figure S3** iPath pathway map of the commonly enriched pathways of differential genes and differential metabolites in the comparison group *rSADS-ΔNS7* vs. *rSADS*. Blue lines in the pathway indicate commonly enriched pathways.

***
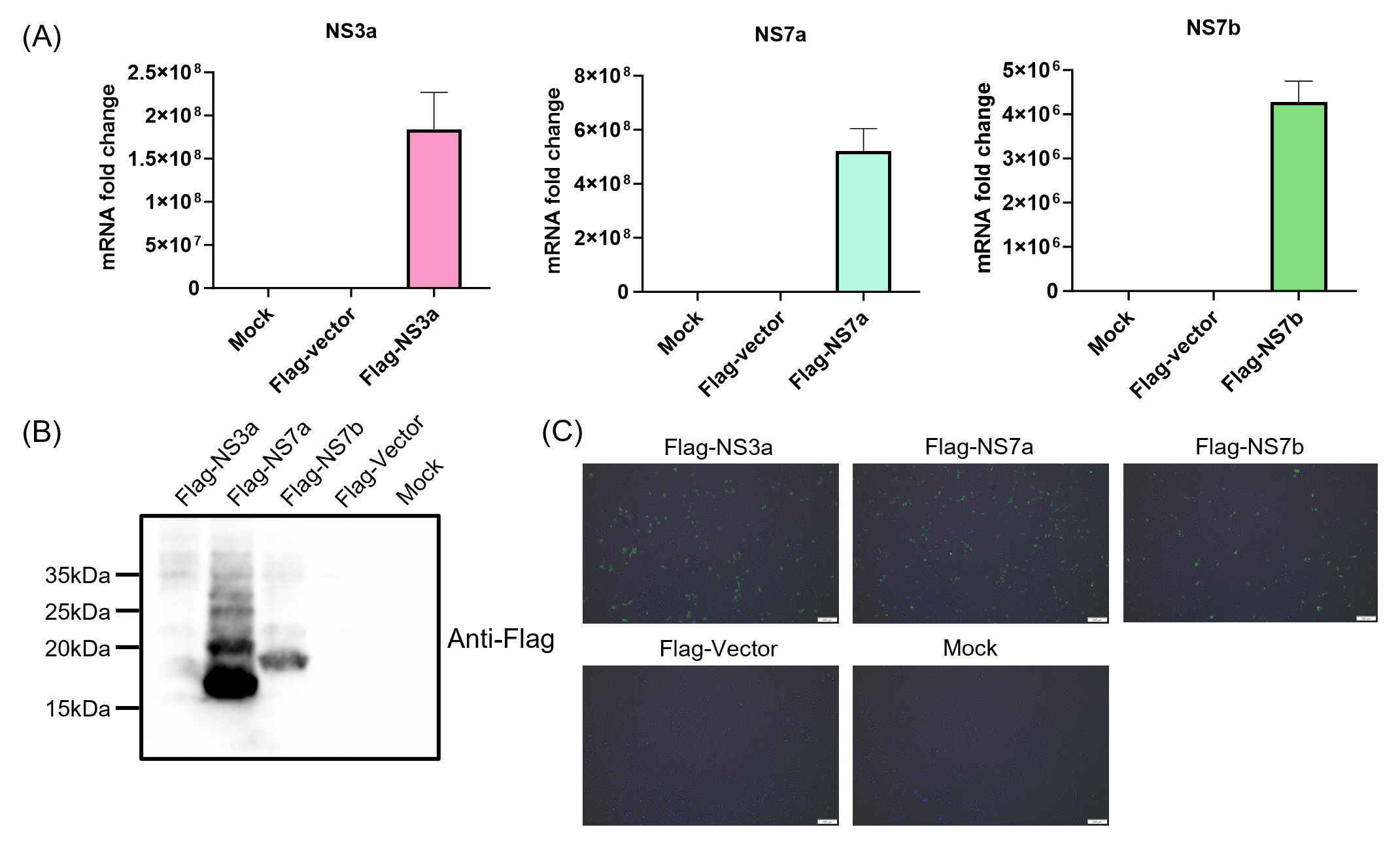
***

**Figure S4** Validation of (A) transcriptional and (B, C) protein expression levels of overexpression plasmids post-transfection in IPI-2I cells. (A) qRT-PCR validation of transcriptional levels of the overexpressed *NS3a*, *NS7a*, and *NS7b* genes constructed with Flag tags. Gene expression levels were calculated using the 2^-ΔΔCt^ method and *β-actin* as an endogenous reference. The Flag-Vector group was used as a control group, and the Mock group was untreated cells. (B) Western blot and (C) IFA validation of expression levels of the Flag-tagged proteins.

***
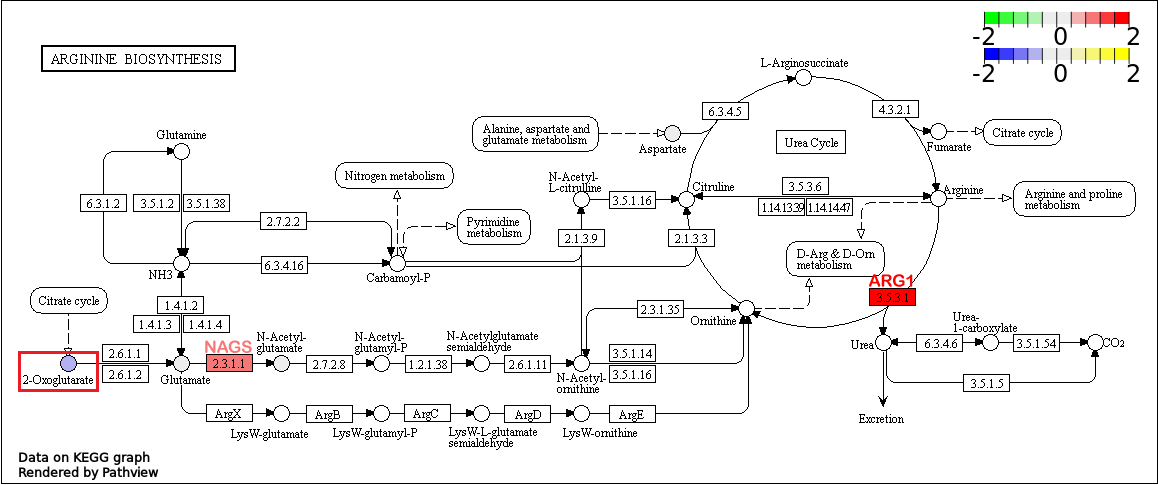
***

**Figure S5** Pathview enriched pathway map showing DEGs and differential metabolites in the arginine biosynthesis pathway for the comparison group *rSADS-ΔNS7* vs. *rSADS*. 2-Oxoglutarate i.e. α-ketoglutarate.

**Table S1** Primer sequences for amplification of DNA fragments required for construction of *NS3a*/*NS7a/b* deletion recombinant plasmids and eukaryotic expression vectors.

| Primer name | Primer sequence (5’-3’)* |
| --- | --- |
| ΔNS3a(all)-ampccdB-F | ACTTCAATCATACGAGATTGAAAAGGTCCACGTCCAATAAGAAACTCAACTAAACTATGTTTCTGAAGATTGTTGAGGATGGCGCGCCTCAAGAAGATCCTTTGATCT |
| ΔNS3a(all)-ampccdB-R | ATCCTCAACAATCTTCAGAAACATAGTTTAGTTGAGTTTCGGCGCGCCGGTGTGGTAGCTCGCGTATT |
| ΔNS7a/b-58bp-ampccdB-F | AAAATCTACCCAGATTTCTTGAGCAAGTCTCGGCTTACTCTAAACCCAGTGGCGCGCCTCAAGAAGATCCTTTGATCT |
| ΔNS7-ampccdB-R | TAGGACTCAATATAATTATACTCTACTCATACTAGCATAAGGCGCGCCGGTGTGGTAGCTCGCGTATT |
| ΔNS7a/b-58bp-F | AAAATCTACCCAGATTTCTTGAGCA |
| ΔNS7a/b-58bp-R | AAACAAATCTGCAAAATCTGCCAAC |
| Flag-NS3a-F | TCATCATTTTGGCAAAGAATTACCCGCCGCCACCATGGCCGACTACAAAGACCATGACGGTGATTATAAAGATCATGACATCGATTACAAGGATGACGATGACAAGATGTTTGGTGGACTTTTCCA |
| NS3a-R | TGACACTATAGAATAGGGCCCTCTAGATGCATGCTCGAGCTCAACAATCTTCAGAAACATAGTT |
| Flag-NS7a-F | TCATCATTTTGGCAAAGAATTACCCGCCGCCACCATGGCCGACTACAAAGACCATGACGGTGATTATAAAGATCATGACATCGATTACAAGGATGACGATGACAAGATGAACCAGCTTATCTTTTT |
| NS7a-R | AAAATCTACCCAGATTTCTTGAGCAAGTCTCGGCTTACTCTAAACCCAGTGGCGCGCCTCAAGAAGATCCTTTGATCT |
| Flag-NS7b-F | TCATCATTTTGGCAAAGAATTACCCGCCGCCACCATGGCCGACTACAAAGACCATGACGGTGATTATAAAGATCATGACATCGATTACAAGGATGACGATGACAAGATGCTATTTTTTTTGATTGGC |
| NS7b-R | TGACACTATAGAATAGGGCCCTCTAGATGCATGCTCGAGCTTACGTGCTTACCATTGTGTATG |
| Flag-F | TCATCATTTTGGCAAAGAATTACCCGCCGCCACCATGGCCGACTACAAAGACCATGACGGT |
| Flag-R | TGACACTATAGAATAGGGCCCTCTAGATGCATGCTCGAGCTTACTTGTCATCGTCATCCTTGTAATCGATGTCATGATCTTTATAATCACCGTCATGGTCTTTGTAGTC |

* Restriction enzyme sites used for cloning are underlined.

**Table S2** PCR validation primer sequences for *SADS-CoV* *NS3a* and *NS7a/b* sites.

| Primer name | Primer sequence (5’-3’) |
| --- | --- |
| SADS-A25-F | GCTCATAACGTCTCTAACATGCG |
| SADS-A25-R | GCATGGTTCAACTTCCATATAAGAC |
| SADS-A27’-F | CAAGAAGCAGAGCTGTCTCAC |
| SADS-A28-R | TGTGTATCACTGTCAAAGAATC |

**Table S3** Fluorescence quantitation detection primers and probe sequences for *SADS-CoV*.

| Primer name | Primer sequence (5’-3’) |
| --- | --- |
| SADS-qF | CTGACTGTTGTTGAGGTTAC |
| SADS-qR | TCTGCCAAAGCTTGTTTAAC |
| SADS-probe | 6-FAM-TCACAGTCTCGTTCTCGCAATCA-TAMRA-N |

**Table S4** Primer sequences for relative fluorescence quantitative PCR.

| Primer name | Primer sequence (5’-3’) |
| --- | --- |
| IL-11(Pig)-F | CCGCACAGCTGAGAGACAAAT |
| IL-11(Pig)-R | GCCTCAGGTAGGAAAACAGGT |
| PLAU-F | AAACCCTTCACTCCAGCACT |
| PLAU-R | TTGTCGGTACGGATCTTCAG |
| PCK1-F | TCCGACCTTCCTTGACCATC |
| PCK1-R | GCCTCTTGATGACACCCTCT |
| ARG1-qF | TGTCTTCCGTTCAGTAGGTGG |
| ARG1-qR | TACACCAGAGTCCTCCAGCC |
| NAGS-F | GTGAGCACCAAAGAACGGC |
| NAGS-R | GCCAGATAGTCGTCCCGCA |
| LAMB3-F | TGCCCAGGAGGGATTTGAGAG |
| LAMB3-R | ATCTTGACGCTCAGGATCCGG |
| Swine-β-actin-F | TCTGGCACCACACCTTCT |
| Swine-β-actin-R | TGATCTGGGTCATCTTCTCAC |
